# Bumblebees learn foraging routes through exploitation–exploration cycles

**DOI:** 10.1098/rsif.2019.0103

**Published:** 2019-07-10

**Authors:** Jackelyn M. Kembro, Mathieu Lihoreau, Joan Garriga, Ernesto P. Raposo, Frederic Bartumeus

**Affiliations:** 1 Universidad Nacional de Córdoba Facultad de Ciencias Exactas, Físicas y Naturales, Instituto de Ciencia y Tecnología de los Alimentos and Cátedra de Química Biológica, Córdoba, Argentina; 2 Concejo de Invesigaciones Cientificas y Tecnologicas, Instituto de Investigaciones Biológicas y Tecnológicas, Córdoba, Argentina; 3 Centre d'Estudis Avançats de Blanes (CEAB-CSIC), Carrer Cala Sant Francesc 14, 17300 Blanes, Catalonia, Spain; 4 Research Center on Animal Cognition (CRCA), Center for Integrative Biology (CBI); CNRS, University Paul Sabatier—Toulouse III, 31330 Toulouse, France; 5 Laboratório de Física Teórica e Computacional, Departamento de Física, Universidade Federal de Pernambuco, 50670-901 Recife, Pernambuco, Brazil; 6 CREAF, Centre de Recerca Ecològica i Aplicacions Forestals, 08193 Bellaterra, Catalonia, Spain; 7 ICREA, Institut Català de Recerca i Estudis Avançats, 08010 Barcelona, Catalonia, Spain

**Keywords:** trapline foraging, bumblebees, t-Stochastic Neighbouring Embedding, movement ecology, exploration–exploitation trade-off

## Abstract

How animals explore and acquire knowledge from the environment is a key question in movement ecology. For pollinators that feed on multiple small replenishing nectar resources, the challenge is to learn efficient foraging routes while dynamically acquiring spatial information about new resource locations. Here, we use the behavioural mapping t-Stochastic Neighbouring Embedding algorithm and Shannon entropy to statistically analyse previously published sampling patterns of bumblebees feeding on artificial flowers in the field. We show that bumblebees modulate foraging excursions into distinctive behavioural strategies, characterizing the trade-off dynamics between (i) visiting and exploiting flowers close to the nest, (ii) searching for new routes and resources, and (iii) exploiting learned flower visitation sequences. Experienced bees combine these behavioural strategies even after they find an optimal route minimizing travel distances between flowers. This behavioural variability may help balancing energy costs–benefits and facilitate rapid adaptation to changing environments and the integration of more profitable resources in their routes.

## 1. Introduction

Any search process, whether in space or mind, individual or collective, involves trade-offs between exploiting known opportunities and exploring for better options elsewhere [1–3]. The optimal balance between exploitation and exploration depends on the specificity of prior information and the quality of current observations [4]. How animals handle the exploitation–exploration trade-off for spatial decisions has broad implications for our understanding of sampling strategies and foraging behaviour. This question has received considerable theoretical interest [1–3] but has rarely been addressed experimentally due to the challenge of acquiring and analysing long-term individual movement data in the field.

Some of the most intriguing animal foraging patterns can be observed in pollinators (orchid bees [5], bumblebees [6], honeybees [7], hummingbirds [8]) and some frugivorous vertebrates (monkeys [9], bats [10]) that exploit replenishing food resources scattered from a central nest or resting site. These animals often visit familiar feeding locations in predictable sequences known as ‘traplines’ [6]. A striking example is the case of bumblebees that collect nectar along traplines minimizing overall travel distances between multiple feeding locations (e.g. flower patches, plants, trees) and the nest [11], a challenging optimization task analogous to the travelling salesman problem in graph theory [12].

Various heuristics have been proposed to explain this routing behaviour, from very simple rules of thumb (e.g. moving between nearest-neighbour flowers [13]) to more sophisticated learning algorithms (e.g. iterative improvement [14]). Only recently have studies acknowledged the non-stationary dimension of search processes [1], trapline foraging being a particular case where it has been shown that trial-and-error and stochastic search rules are an essential part of route learning and stabilization [11,15,16]. Bumblebees may always maintain an exploratory behavioural component even after fixing movement transitions between particular flowers [16] or a complete route [11]. Presumably this continuous exploratory strategy allows for rapid adoption of new efficient routes following environmental perturbation [17]. Bees are known to display distinct spatial behaviours attributed to different navigational functions [18], such as learning flights [19,20] and orientation flights [19] to acquire view based memories of important locations (e.g. feeding sites and nest) on their first foraging attempts, route-following to link familiar locations [20] and search loops when some of these locations are experimentally moved or when bees are displaced in new areas [21,22]. While these movement patterns have been described in experimental scenarios specifically designed to study each strategy individually, how different behaviours are dynamically unfolded and used by bees during the process of route formation is unknown.

To understand how a bee combines these behavioural strategies to sample flowers, and exploit vast, fragmented and fast-changing foraging areas, we need to precisely measure and characterize behavioural patterns. Part of the problem arises from the need to specify a key behavioural descriptor (e.g. transition probabilities between flowers, flight times, revisit frequencies) and finding the adequate spatio-temporal scale of observation that accounts for meaningful behavioural sequences. For instance, in the two studies that provide high-resolution data on trapline formation by bumblebees, Lihoreau *et al*. [23] quantified exploration based on the visual identification of flight loops starting and ending at the same feeding location, whereas Woodgate *et al*. [16] considered flights in which bees, starting at the vicinity of a flower, flew more than 50 m from all previously discovered flowers. While both metrics may ultimately reveal some tendency for exploration, the definition radically differs, affecting our views about how animals produce and organize motor patterns.

Unsupervised machine learning clustering methods (see overview in the electronic supplementary material, section S1) offer the possibility to statistically map behavioural high-dimensional spaces based on minimum assumptions [24]. While this approach has been increasingly used to identify common behavioural principles across taxa, and describe the modularity and hierarchical organization of behaviour in laboratory set-ups using model organisms such as *Caenorhabditis elegans* [1,25,26], *Drosophila melanogaster* [27,28] or zebrafish [29], it also holds considerable promises for analysing complex movement data from field studies, accounting for a much more principled behavioural classification and avoiding arbitrary binary descriptions.

Here, we used an unsupervised l mapping method based on the t-Stochastic Neighbouring Embedding (t-SNE) algorithm [24,27,30,31] to characterize the spatial behaviour of bumblebees (*Bombus terrestris*) foraging in semi-field conditions. The t-SNE is a dimensionality reduction machine learning method suited to identify repetitive multivariate patterns (or stereotypical sequences) in the dataset (see electronic supplementary material, section S1). The data (previously published by Lihoreau *et al*. [23]) included all flower visitation sequences by focal bumblebees using motion detection cameras placed on five artificial flowers arranged in a regular pentagon with 50 m sides during a full day. Each flower contained a sucrose solution reward equivalent to one-fifth of the crop of the bumblebee (stomach) capacity and was refilled after each foraging bout (foraging trip starting and ending at the nest entrance), in order to encourage route development between all flowers. Herein, we analysed all foraging bouts with global statistical descriptors to build a behavioural landscape partitioned into significant domains to identify elementary and repetitive types of foraging bouts. We then classified foraging bouts into specific behavioural strategies, and characterized behavioural variability in terms of Shannon entropy and flight time probability distributions. Using this approach, our aims were (i) to identify exploration–exploitation behavioural strategies throughout the process of route formation and (ii) to investigate behavioural variability and its potential association with effective stochastic search and trial-and-error learning.

## 2. Results

### 2.1. Three main behavioural strategies characterize the variability of foraging bouts

**Figure 1.**
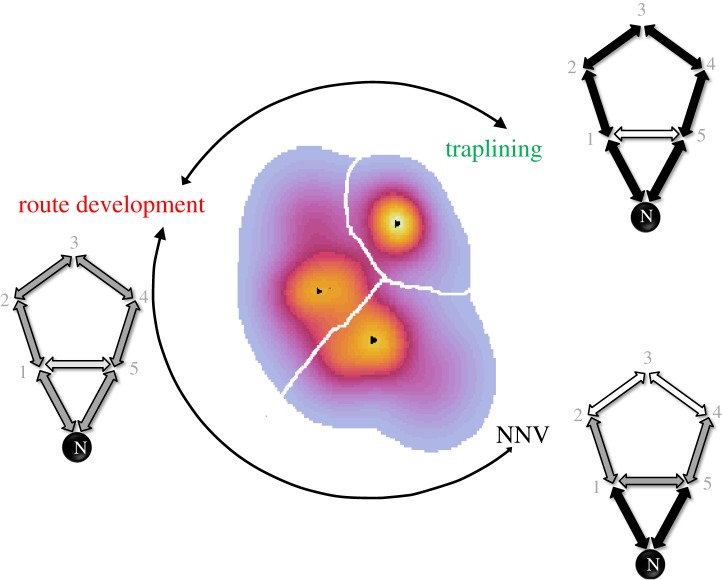
Behavioural landscape. Quantitative analysis of bumblebee foraging bouts in a large-scale set-up containing the nest-box (N) and 5 artificial flowers (1–5). Computation of the behavioural landscape of foraging bouts from seven bees based on a t-Stochastic Neighbouring Embedding (t-SNE) analysis. The heat map of t-SNE landscape reveals three behavioural strategies: NNV, Route Development and Traplining. The dominant behavioural mode characterizing each strategy is defined by a peak (black dot). The smaller the size of the clustered region and the larger the strength of the dominant peak, the less variable the foraging behaviour. The largest clustered area and the lowest peak correspond to the Route Development strategy, whereas the smallest clustered region and strongest dominant peak are associated with the Traplining strategy. Skeleton diagrams show representative foraging bouts (data from all bees pooled together) for each strategy. Only transitions between near flowers are represented for simplicity. White, dark grey and black double arrows represent transitions that occurred 0%, greater than or equal to 4% and greater than or equal to 7% of foraging bouts of each type, respectively. Grey numbers denote each flower. We used 11 variables for t-SNE analysis (table 1 and figure 2*a*): length of the flower visitation sequence, probability of immediate revisits to a flower, numbers of different flowers found and probability of visiting the flower furthest from the colony nest (flower 3), probability of symmetrical 2-flower transition types 3, 4 and 5 (electronic supplementary material, figure S20), probability of 4-flower transition type 1 and 2, probability of 5-flower transition and the determinism index. (Online version in colour.)

Our characterization of the bumblebee flower visitation sequences defined a behavioural landscape delimited by three statistically significant domains (figure 1). Each domain represented a dominant (and differentiated) movement mode, suggesting distinct behavioural strategies that we name: Near-nest visits (NNV), Route Development, and Traplining (figure 1 and table 1). The Route Development strategy showed the largest and flattest area of the behavioural space (figure 1), representing a highly variable behaviour. The Traplining strategy involved a much smaller area, with a narrow and high peak (figure 1) indicative of highly repeatable and predictable behaviour. The NNV strategy resulted in an intermediate peak size and area coverage (figure 1), indicating both some level of variability and repeatability. There is of course some relationship between the shape of the t-SNE behavioural landscape and the observation window used to characterize route development and learning processes by bumblebees. However, here it is important to recall that a predominance (peak) in the t-SNE landscape depends on the relative similarities of the parametrized flower visitation patterns rather than on the frequency of the different patterns observed along the learning process.

**Table 1.** Behavioural variables used in t-Stochastic Neighbouring Embedding (t-SNE) analysis for determining Near-nest visits (NNV), Route Development and Traplining behavioural strategies. Note: Ten out of the 11 variables for t-SNE analysis are shown. The determinism index is depicted in electronic supplementary material, figure S2A.

	NNV	Route Development	Traplining	*p*-value
length of the flower visitation sequence	6^a^ (5; 7)	8^b^ (6; 13)	7^b^ (7; 7)	<0.0001
immediate revisits (%)	0^a^ (0; 20)	14^b^ (0; 24)	0^c^ (0; 0)	<0.0001
number of flowers found	4^a^ (3; 5)	5^b^ (5; 5)	5^b^ (5; 5)	<0.0001
probability of visiting farthest flower from the nest (%)	0^a^ (0; 0)	20^b^ (14; 27)	14^c^ (14; 14)	<0.0001
symmetrical 2-flower transition of type 3 (%)	0^a^ (0; 0)	14^b^ (8; 20)	17^b^ (17; 17)	<0.0001
symmetrical 2-flower transition of type 4 (%)	0^a^ (0; 0)	11^b^ (0; 14)	17^c^ (17; 17)	<0.0001
symmetrical 2-flower transition of type 5 (%)	0^a^ (0; 17)	0^b^ (0; 8)	17^c^ (17; 17)	<0.0001
4-flower transition type 1 (%)	0 (0; 0)	0 (0; 06)	25 (22; 25)	—
4-flower transition type 2 (%)	0 (0; 0)	0 (0; 0)	25 (25; 25)	—
5-flower transition (%)	0 (0; 0)	0 (0; 0)	25 (22, 25)	—

Median (Q1; Q3)

^a–c^Strategies that do not share the same letter differ by *p* < 0.05 (Kruskal–Wallis).

**Figure 2.**
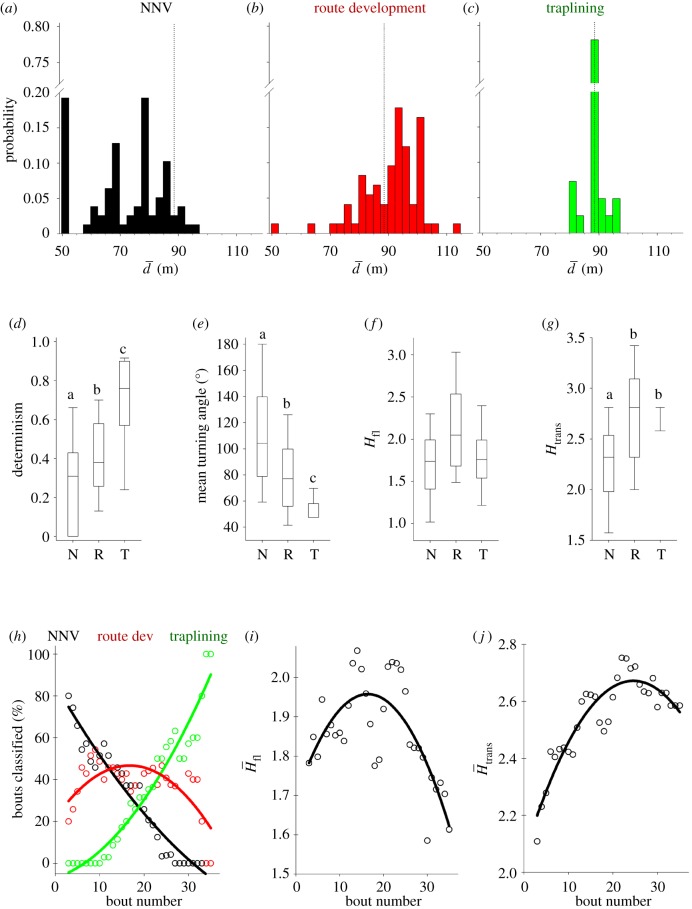
Characterization of behavioural strategies and their unfolding across time. (*a*–*c*) Probability distribution of the mean distance to the nest (*d*) estimated as the mean distances expressed in metres between each flower visited during a foraging bout (including revisits) and the nest. Distinct distributions are observed in NNV, Route Development and Traplining behavioural strategies. Dotted black line represents an optimal trapline. (*d*–*g*) Boxplots show foraging bout variability of four variables for Near-nest visits (N), Route Development (R) and Traplining (T). (*d*) Determinism estimated for each period of three consecutive foraging bouts, and a minimum designated series length of three flowers, (*e*) mean turning angle (i.e. the mean of the absolute values of turning angles) and (*f*,*g*) Shannon entropies (*H* = −Σ*p*(*x*)log_2_*p*(*x*)), *H*_ft_ where *p* is the relative flight duration (i.e. flight duration/duration of foraging bout), and *H*_trans_ where *p* is the probability of performing one of the 36 possible transitions between flowers and/or the nest. (*d*–*g*) Strategies that do not share the same letter differ by *p* < 0.05 (Tukey and Kramer (Nemenyi) test with Tukey-Dist approximation for independent samples). (*h*) Per cent of foraging bouts classified as belonging to each behavioural strategy as a function of the temporal sequence of bouts, computed over five foraging bouts overlapped running windows. Lines show a quadratic polynomial fit to data points of each foraging bout class. (*i*,*j*) Median values of entropies *H*_ft_ and *H*_trans_ for all seven bees, computed over five foraging bouts overlapped running windows. For illustrative purposes, we show quadratic polynomial fits to data points as lines. (Online version in colour.)

Skeleton diagrams illustrate the principal differences between the three behavioural strategies (figure 1). In the foraging bouts classified as NNV, bumblebees visited flowers closer to the nest (figure 2*a*) in comparison to the other two strategies (figure 2*b*,*c*). These foraging bouts also showed lower levels of determinism (i.e. a measure of route repeatability [32], figure 2*d*), and larger mean turning angles (i.e. the mean of the absolute values of angles between three successively visited flowers) in comparison to the other two strategies (figure 2*e*). In NNV bouts, bees made seldom transition towards the flower furthest from the nest (table 1), and on average half of the transitions were performed towards nearest-neighbour flowers (table 2). In the Route Development foraging bouts, bumblebees made more flower visits (table 1; electronic supplementary material, figure S17), travelled longer mean distances from the nest (figure 2*b*) and showed an intermediate mean turning angle (figure 2*e*) in comparison to the other two strategies. Bumblebees also made a larger number of nearest-neighbour transitions than the NNV strategy (table 2). Finally, in the Traplining bouts, bumblebees performed almost exclusively near-neighbour transitions (table 2). Individuals showed a high probability of performing three- or four-flower symmetrical transitions towards nearest neighbours (note that these were not performed in the other two behavioural strategies, table 1). Consequently, bumblebees had significantly higher determinism and lower mean turning angles (figure 2*d*,*e*). Considering that determinism was estimated for three consecutive foraging bouts, the high value observed for Traplining bouts indicated repetition of the same pattern in consecutive foraging bouts, which is also reflected by the low variability observed in all the variables estimated (figure 2 and electronic supplementary material, figure S17, tables S1 and S4).

**Table 2.** Key variables associated with behavioural strategies in bumblebees as determined by t-Stochastic Neighbouring Embedding (t-SNE) analysis (i.e. Near-nest visits (NNV), Route Development and Traplining).

	NNV	Route Development	Traplining	*p*-value
nearest-neighbour transitions (%)	54^a^ (40; 67)	60^a^ (44; 75)	100^b^ (91; 100)	<0.0001
time visiting flower (s)	82^a^ (52; 130)	110^b^ (77: 149)	145^c^ (108; 183)	<0.0001
per cent of bout time visiting flower (%)	20^a^ (11; 33)	24^a^ (12; 33)	40^b^ (32; 49)	<0.0001
time visiting near-nest flowers (s)	45^a^ (25; 79)	29^b^ (22; 55)	44^a^ (37; 72)	0.01
per cent of visits to near-nest flowers (%)	60^a^ (50; 80)	33^b^ (25; 50)	40^b^ (40; 40)	<0.0001
time flying/time visiting	5.5^b^ (2.2; 11.7)	3.8^b^ (2.2; 9.1)	2.0^a^ (1.2; 4.4)	<0.0001
bout duration/number of different flowers	155^a^ (102; 243)	114^b^ (84; 197)	66^c^ (57; 100)	<0.0001

Median (Q1; Q3)

^a–c^Variables that do not share the same letter differ by *p* < 0.05 (Kruskal–Wallis test).

Bumblebees performing the Traplining strategy maintained an average distance from the nest during flower visits of approximately 89 m in 78% of their foraging bouts (figure 2*c*), which is equal to the average distance obtained during an optimal sequence minimizing overall travel distances (N–1–2–3–4–5–N or N–5–4–3–2–1–N, figure 1). The average distance from the nest (figure 2*a*–*c*) can be considered an indicator of the travelling costs. In the NNV strategy, 91% of the foraging bouts were characterized by a mean distance from nest of less than the optimal 89 m (figure 2*a*, dotted black line), reflecting a low-cost type of strategy. Accordingly, the Route Development strategy is the one showing a higher cost compared with the other two, with 68% of the bouts at a mean distance from nest beyond the optimal 89 m (figure 2*b*).

Bumblebees spent more time on flowers in the Traplining strategy than in the Route Development or NNV behavioural strategies (table 2). They also showed a larger number of different flowers visited and the lowest ratio between time flying and time visiting a flower (table 2), a signature of flower exploitation. In Route Development bouts, bumblebees spent less time on the two flowers nearest to the nest (flowers 1 and 5, figure 1) in comparison to the other two strategies (table 2). In NNV bouts, bees showed a higher proportion of time on near-nest flowers relative to total bout time compared to the two other strategies (table 2). Interestingly, bumblebees in NNV bouts showed a higher ratio between bout duration and the number of different flowers visited (cost of flying/reward) in comparison to the other strategies, while intermediate values were observed in the Route Development strategy (table 2). These results suggest that bumblebees executing the NNV strategy explore their environment but preferentially exploit flowers near the nest. However, we cannot exclude the possibility that bumblebees also use exploration routes undetectable with flower visitation sequence data, such as, for instance, long convoluted flight paths between visiting flowers.

At the population level (i.e. when each of the foraging bouts of each of the seven bumblebees are pooled together), a clear time sequence is observed in the predominance of each of the three strategies (figure 2*h*, see also arrows in figure 1), despite time not being made explicit in the t-SNE analysis. NNV bouts occurred predominately (approx. 70%, black line figure 2*h*) at the beginning of the learning process and decreased in frequency with experience. Traplining bouts started to appear after bout 10 (red line figure 2*h*). Interestingly, Route Development bouts co-occurred with the other two strategies, remaining relatively constant in percentage (between approx. 20 and 50%, green line figure 2*h*) across time. At intermediate times (bouts 15–22, figure 2*h*), the percentages of both NNV and Traplining bouts were lower than the percentage of Route Development bouts. Thus, the percentage of Route Development bouts became relatively large compared with the other two types of behavioural strategies at intermediate times.

### 2.2. Route development bouts lead to larger behavioural richness and entropic disorder

For each behavioural strategy, we computed the Shannon information entropy (Materials and methods, equation (4.1)) as an indicator of the level of uncertainty and the amount of degrees of freedom associated with flight time durations between flower visits (*H*_fl_) and pairwise transitions between flowers (*H*_trans_). In general, *H*_ft_ was significantly higher during Route Development bouts compared to NNV and Traplining bouts (figure 2*c*), which is consistent with the larger variability observed in flight times (electronic supplementary material, figure S13B) and the longer flower visitation sequences (electronic supplementary material, figure S13D). *H*_trans_ also increased in Route Development bouts in comparison with NNV bouts, but not compared to Traplining bouts (figure 2*d*). These results are concordant with the observation of longer flower visitation sequences (electronic supplementary material, figure S14*a*) and higher number of different flowers visited per bout (electronic supplementary material, figure S14*b*) for Route Development bouts compared to NNV and Traplining bouts. At the beginning of the experiment (when NNV bouts were prevalent), foraging bouts showed low entropy values (figure 2*i*,*j*). These values increased at an intermediate stage of the experiment (when Route Development bouts were prevalent). At the end of the experiment (when Traplining bouts were prevalent) *H*_ft_ was lower and *H*_trans_ was somewhat stabilized. Noteworthy, although calculations of *H*_fl_ and *H*_trans_ are based on different variables, flight times and pairwise flower transitions, respectively, a significant positive correlation exists between the two (*y* = 1.175 + 0.697*x*; *r*^2^ = 0.574). All in all, the entropic perspective suggests that NNV and Traplining strategies are more exploitative compared to the Route Development strategies that shows a stronger exploratory component. The entropic analysis is also concomitant with the fact that, in the t-SNE behavioural landscape (figure 1) the Route Development strategy is characterized by a flatter and much larger area than the NNV or Traplining strategy, suggesting the unfolding of a much richer behavioural repertoire and larger behavioural degrees of freedom at intermediate stages of the learning process.

### 2.3. Exploitation–exploration dynamics is more complex at the individual than at the population level

**Figure 3.**
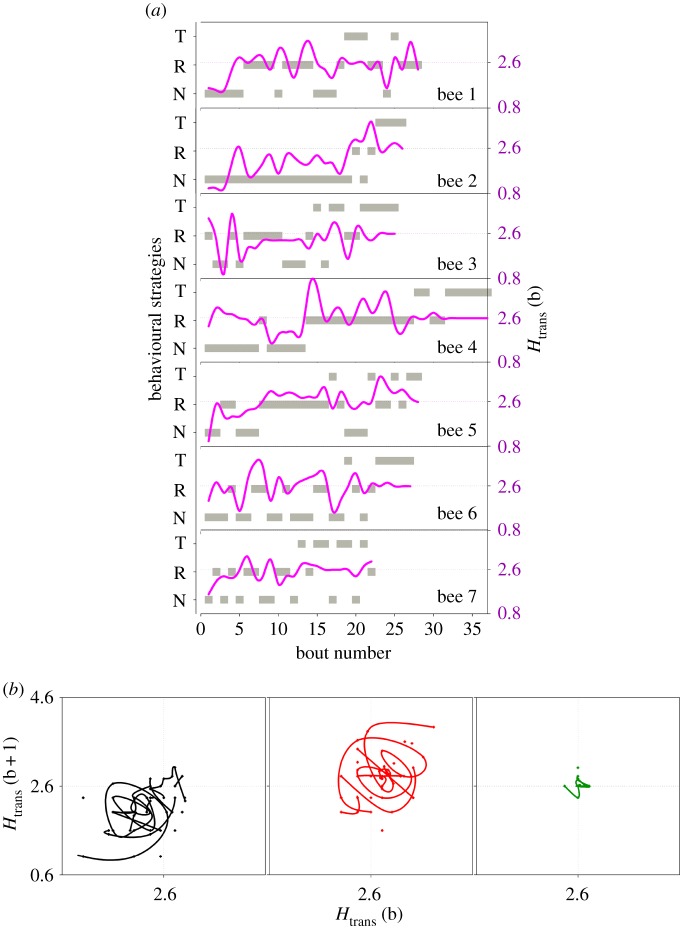
Temporal evolution of behavioural strategies and entropy from naive to experienced bumblebees. (*a*) Time series of fluctuations between behavioural strategies Near-Nest Exploitation (N or NNV), Route Development (R) and Traplining (T) throughout route learning of each of the seven bumblebees evaluated. Grey squares represent the behavioural strategy (left axis) and lines represent the estimated entropy *H*_trans_ (right axis) of given bout. (*b*) Return plots of entropy *H*_trans_ estimated in two successive foraging bouts of the same behavioural strategy. Note the oscillatory nature of *H*_trans_ during NNV and Route Development bouts where higher values are often followed by lower entropies. Similar results are shown for *H*_fl_ in electronic supplementary material, figure S15. Moreover, NNV bout oscillations occurred mainly between 0.8 and 2.59, while between Route Development bouts a larger variability was observed mainly between 1.7 and 4.2. By contrast, during Traplining fairly constant values of *H*_trans_ were observed near the optimum value 2.6 (dotted lines). (Online version in colour.)

Although, on average, foraging patterns showed an ordered sequence of behavioural strategies, each bumblebee did not perform a completely ordered set of behavioural transitions. On the contrary, a complex sequence of behaviours was observed through time (figure 3*a*; electronic supplementary material, figures S15 and S18). For example, towards the end of the experiment (after foraging bout 22) individual five performed the sequence: Traplining (T)–Route Development (R)–NNV (N)–T–R–T–T (figure 3*a*). Transitions through the t-SNE space are erratic (electronic supplementary material, figure S18), suggesting a non-deterministic learning process and competing drives for exploration and exploitation, i.e. bumblebees balance the decision to continue exploiting close-by flowers from the nest, performing an optimal trapline, or seeking for other potential food sources.

In addition to alternating between distinct behavioural strategies, within a given strategy, individual bumblebees showed periodic fluctuations in entropy levels, both *H*_trans_ and *H*_fl_ (figure 3*a*; electronic supplementary material, figure S15A). Most evidently, in the NNV and Route Development strategies, large entropies were frequently followed by low entropies, suggesting that bumblebees alternated excursions where they visited large and diverse sets of flowers with much simplified foraging bouts involving small and less diverse sets of flowers (electronic supplementary material, figure S14A,B,D,E).

**Figure 4.**
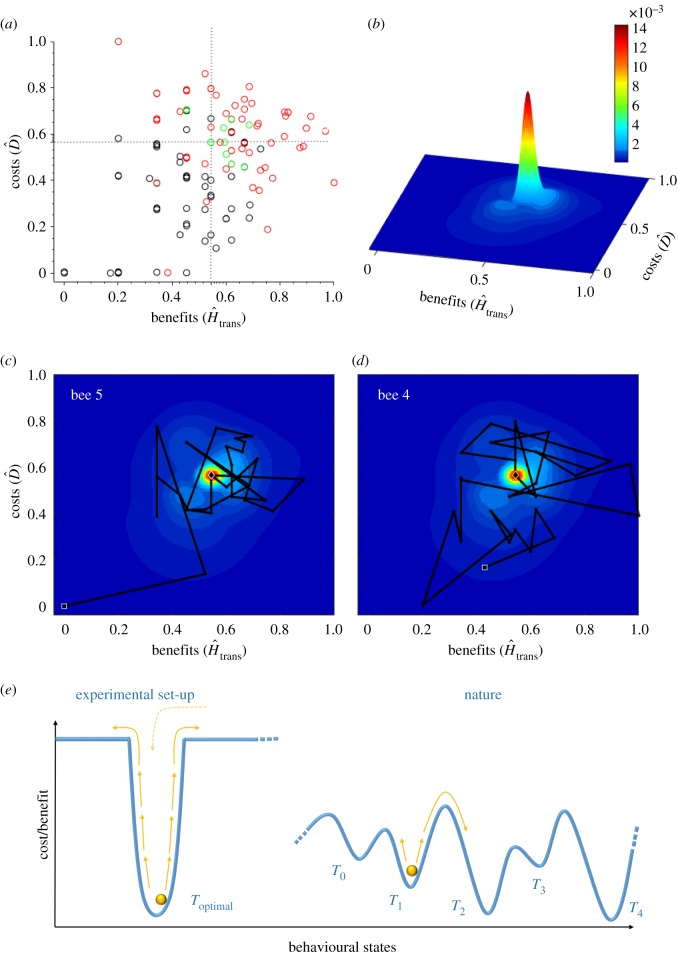
Behavioural transition from naive to experienced bumblebees depicted in a cost–benefit landscape, and contextualized in simple and complex fitness landscapes for optimal routing. (*a*) The three behaviourally distinct strategies of figure 1 depicted in a cost–benefit landscape (distance from nest $\hat{D}$ versus exploratory activity ${\hat{H}}_{\mathrm{trans}}$). Near-Nest Exploitation (NNV, black open circles) represents low distances from nest and low exploratory activity, whereas Route Development (red open circles) represents large costs associated with being far from the nest and large exploratory activity relative to Traplining (green open circles). (*b*) Three-dimensional representation of a two-dimensional kernel density estimation of the population-level frequency of foraging bouts with a given amount of cost–benefit exposure (data of all bees pooled together). The peak represents optimized trapline foraging behaviour. Note also the smaller plateaus in the lower-left and upper-right quadrants, representing opposite behavioural strategies, surrounding optimal trapline foraging. (*c,d*) Examples of two-dimensional kernel density population-level representations with the individual sequences of foraging bouts for two different bees. (*e*) The different possible behavioural states an animal can adopt can be associated with a cost/benefit ratio. In this context, an optimal trapline minimizing travel distances can be associated with a local minimum of a cost/benefit. Attractors (local cost/benefit minimums) are constantly perturbed due to environmentally driven or induced behavioural variability, that may come from imperfect memory or motor control at some level, casting the forager out of the local minimum to another (potentially better) minimum. Depending on the overall behavioural state landscape dynamics can be stable or unstable attractor points, so that bees may end up returning to specific spatial configuration (as is the case of the experimental set-up) or else never come back, a process endured by a never-ending exploratory process only constrained by the distance to the nest. (Online version in colour.)

Although noisy, this oscillatory dynamics (electronic supplementary material, figure S19) was also evidenced in return plots (figure 4*b* and electronic supplementary material, figure S15B), where the value of entropy estimated at a given foraging bout (b + 1) was plotted as a function of the previous foraging bout (b) of the same behavioural strategy. In the case of NNV bouts, oscillations in *H*_trans_ occurred mainly in the range of 0.8 and 2.6 (left quadrant in figure 3*b*), while in Route Development bouts a larger variability was observed and values ranged mainly between 1.7 and 4.2 (middle quadrant in figure 3*b*, centred around the optimal value 2.59). By contrast, in Traplining bouts we observed almost constant values of *H*_trans_ close to the optimum value 2.59 (figure 3*b*). Similar results were observed in return plots for *H*_fl._ Nonetheless, for the later entropy Traplining bouts showed a much wider range of values in the return plot compared to *H*_trans_ (electronic supplementary material, figure S15B). Therefore, within each behavioural strategy bumblebees showed low–high variability cycles in terms of both flower transitions and flight durations between visits. Only in the Traplining strategy, flower transitions did not show this pattern as they were mostly fixed.

Following [33], we extended our entropy analysis to study routine movement behaviour across time by estimating the pth-order conditional entropy, *H*_p_. Similar to the idea of determinism [32], ‘routine movement behaviour’ involves some degree of regularity in the movement sequence pattern, which can be defined as the opposite of uncertainty. Our results (electronic supplementary material, figure S21) are consistent with the other estimators used in this work (i.e. Shannon entropy, determinism), the first-order conditional entropy values fluctuate at the short-time scale, but decrease throughout route learning. The short-scale fluctuations suggest that bumblebees alternate the exploitation of repeatable flower visit sequences with the exploration of new sequences, despite on average routine movement behaviour increasing steadily with time. In any case, the routine movement behaviour analysis should be taken cautiously due to limitations in data structure and length (see caption at electronic supplementary material, figure S21).

Finally, we also analysed the two-dimensional flight trajectories of bumblebees in some of the foraging bouts where individuals were also tracked with a harmonic radar (electronic supplementary material, §3). The results suggest that different flower-sampling behaviours are used through time, as bumblebees gained experience on the experimental system (electronic supplementary material, §3).

## 3. Discussion

We used an unsupervised mapping approach to characterize and analyse behavioural strategies of bumblebees while developing foraging routes between multiple resources in a large semi-field set-up and show that bumblebees develop routes by alternating exploration (Route Development) and exploitation (NNV and Traplining) cycles. Trial–error excursions (Route Development) were alternated with the exploitation of resources near the nest (NNV) and, in more advanced stages, with the utilization of stable multi-location routes (Traplining). Radar data analysis (electronic supplementary material, §3) also reveals fine-grained behavioural changes by bumblebees when spreading out from flowers as experience progresses. This result reinforces the idea that different flower-visiting strategies are unfolded as bumblebees retain information about flower location and potential optimal routes. Our approach shows that foraging bout variability is naturally grouped, according to bumblebee behavioural signatures, in three possible strategies. Our method signifies behavioural stereotypes and avoids unnecessary data pooling or arbitrarily grouping often used in comparative behavioural analyses. In the NNV strategy, bumblebees mainly performed strong turning angles, as a way to restrict their initial foraging bouts in space, and by revisiting nearby subsets of flowers, which were known to be rewarding. This pattern may simply emerge because bumblebees take time to find flowers and acquire knowledge of their spatial locations. Although in the NNV strategy resource exploitation near the nest is prevalent, some excursions to flowers far from the nest were also observed, consistent with Woodgate *et al.* [16]. The Route Development strategy showed larger behavioural degrees of freedom (i.e. t-SNE area), and a larger behavioural entropy in flower transitions and flight times between visits in comparison to the other two strategies. Therefore, while exploring, bumblebees are able to locate all the flowers and stabilize relevant routes at the cost of using more variable and suboptimal (long) paths. Albeit being potentially highly energetically costly [34,35], a stronger variability in behavioural strategies may facilitate trial-and-error learning and improve discovery rates, speeding up convergence to an optimal route. After approximately 10 foraging bouts, the Traplining strategy emerged, involving repetitive and ordered visitation sequences to all the flowers in the array. This strategy was characterized by a strong and localized peak in the behavioural landscape (figure 1).

Nearest-neighbour transition rules (i.e. systematic movement to the nearest unvisited target) have often been suggested as a relevant behavioural mechanism for route development and fixation by bees [16,36] (but see [11]). Indeed, in the NNV and Route Development strategies, nearest-neighbour transitions represent about half of the transitions between two flowers (see Symmetrical transitions in electronic supplementary material, table S4). This result is consistent with the observations of Woodgate *et al.* [16] in a different array of flowers where nearest-neighbour transitions always led to suboptimal routes, which was not the case in our regular pentagonal array of flowers. Therefore, experiments involving more complex spatial configurations of flowers in which nearest-neighbour transitioning does not necessarily lead to short routes (e.g. [11,16]) are needed to clarify the importance of the nearest-neighbour behaviour for route formation and optimization.

At the population level, a clear temporal sequence from NNV to Route Development and Traplining strategies emerged, with Route Development essentially co-occurring through time, especially at intermediate training stages (figure 2*h*). At the individual level, however, the strategy transition dynamics was less straightforward. For example, the NNV strategy, although predominating at the beginning, appeared recurrently throughout the route learning process as well, suggesting complex individual decision-making (figure 3 and electronic supplementary material, figure S15; see also the routine movement analysis in electronic supplementary material, figure S21). The Traplining strategy started to appear after foraging bout number 10; however, bumblebees tended to return to Route Development strategies during subsequent foraging bouts. This result is in line with previous observations suggesting that once routes are stabilized, bumblebees often make further foraging bouts with alternative visitation sequences [11,16]. Imperfect learning and memory could favour these back-and-forth behavioural shifts in experienced foragers. These cycles between exploitation and exploration strategies could also reflect an inherent behavioural strategy, that is, the production of controlled motor variability alternating the discovery of new flowers and flower sequences with the exploitation of routes already fixed [16,23]. There are considerable, unexplored connections between the current study and that of Bartumeus *et al.* [1] related to the fact that search is essentially an organized, non-stationary stochastic process, where exploration and exploitation processes of any kind must alternate. Provided that the environment is stable enough for the animal to gain information about it, the learning process itself becomes a strong driver of behavioural change that should be reflected in the motor output, through adjustments between internal states, memory and discovery rates.

To reinforce the idea that stochastic search does not represent fully unstructured noise, we showed that the statistical signatures found for the Route Development strategy are not obtained by adding a simply structured noise to the optimal route (electronic supplementary material, §4). As an example, the addition of Gaussian noise to optimized traplines changed some of our behavioural descriptors according to expectation, as it: (i) increased the variability between foraging bouts (e.g. entropy *H*_trans_ and number of flower visits; electronic supplementary material, figure S16C and S16D, respectively), (ii) decreased determinism, and (iii) increased the turning angles (electronic supplementary material, figure S16A and S16B). However, Gaussian-driven stochasticity could not explain (i) the anomalous diffusion observed in the local spreading out of the flowers, (ii) the large amount of flight durations beyond the characteristic time scales associated with the experimental set-up, nor (iii) the wild heterogeneity (multiple scales) and scaling properties observed on flight time distributions (electronic supplementary material, figure S13).

To visualize the costs and benefits of the overall foraging strategy of bumblebees, we parametrized each foraging bout in terms of the average distance to the nest (cost related to the energy expenditure and error accumulation when flying) and the entropy associated with flower transitions (well correlated with the Route Development activity and the benefit of finding new flowers or routes) relative to optimized traplining behaviour (figure 4). We found that NNV, Route Development and Traplining behavioural strategies can be mapped following a gradient from low cost–low benefit (NNV) to large cost–large benefit scenarios (Route Development; figure 4*a,b*). Transitions from naive to more experienced foraging phases result in very erratic paths in the depicted cost–benefit landscape, finally leading to Traplining (figure 4*c,d*). This reflects the presence of individual trade-offs and alternation of behaviourally distinct sampling strategies, likely in part related to trial-and-error route learning and optimization.

If we consider route learning as a dynamical behavioural system (figure 4*e*), the Traplining strategy would be the main attractor. In our simplified experimental context, where all flowers were equally rewarding and replenished after each foraging bout, the optimal trapline is a stable attractor. In most natural conditions, however, bumblebees may experience much more complex cost–benefit (fitness) landscapes, with highly unstable environments due to variability in resource replenishment rates [37,38], competition [39–41], changing weather conditions [42,43] and the presence of predators [44]. In such conditions, constant exploration might be crucial to track new options and efficiently adjust foraging routes. Hence, traplines may become unstable attractors, subject to constant perturbations both environmentally or behaviourally induced, caused either by imperfect learning or by the need of new discoveries. Our results suggest that departures from specific traplines (local minima) could rely on enhanced spreading dynamics out of particular flowers or traplines, resembling fast simulated annealing, as suggested in Lihoreau *et al*. [15].

Quantifying pollinator behaviour has historically been difficult [45] and long raised the need to develop metrics for describing and comparing their sophisticated spatial patterns (see discussions in [6,32,46]). The use of unsupervised mapping methods on bumblebee data allowed us to quantify emerging flower-sampling strategies and individual behavioural transitions (exploitation–exploration) during route learning, both from a bottom-up approach and at an unprecedented level of detail. Although not entirely free of assumptions, this methodology is statistically robust and better principled (well-grounded on information theory) than previous approaches. Beyond research on pollinators, we believe that such approach will be instrumental for future quantitative research in movement and behavioural ecology, guided by powerful assessments and comparisons of behavioural variability, both structure and dynamics, in field conditions.

## 4. Material and methods

### 4.1. Subjects and study site

The experimental set-up is described in detail by Lihoreau *et al.* [23]. A bumblebee colony was installed in a flat, open area of mown pasture (51.805149° N 0.365080° W) free of natural flowers. The entrance tube of the colony box was equipped with a series of gates in order to control the traffic of foragers. Bumblebees were only allowed to leave the colony during the pre-training and training periods. During pre-training, all foragers could freely explore the outside environment and collect sucrose solution (40% w/w) from five artificial flowers arranged in a linear array (150 cm length) 50 m northwest of the nest entrance. Flowers were refilled ad libitum with 10 μl of sucrose solution to estimate the crop capacity of each bumblebee. Pre-training typically lasted one day. Regular foragers that made at least five foraging bouts in 1 h were selected for testing. During testing, only one forager was allowed to leave the colony. This focal bumblebee was observed foraging on flowers arranged in a regular pentagon (figure 1). The bumblebee was therefore familiar with the outside environment but unfamiliar with the array of flowers. Each flower was at a distance of 50 m from two neighbour flowers, 80 m from the opposite flowers and 108 m from the most distant flower. These distances were chosen to promote exploration. Given that *B. terrestris* workers are able to detect reflecting (non-self-luminant) targets from a background subtending a visual angle of *ca* 3° [47] we assume that bees could detect our flowers from a maximal distance of 13 m from any location in the experimental field. Each flower contained a sucrose reward equivalent to one-fifth of the crop capacity of the focal bumblebee and was refilled after each foraging bout. Testing typically lasted one day. Each bumblebee was tested on different days.

### 4.2. Flower-sampling sequences

Seven individuals were trained in the pentagonal array until they found all flowers and visited them in the same sequence in at least three consecutive foraging bouts (6.7 ± 1.0 (s.e.) hours of observation and 27.6 ± 1.8 foraging bouts per bumblebee). During the tests, all departure and arrival times at the nest were recorded by the experimenter and were considered as all the times in which the bee left and then returned to the colony during testing. Flower visits were recorded using motion-activated webcams (Logitech c250, Fremont, CA) mounted above each flower. Video clips provided arrival and departure times from each flower, from which we reconstructed the flower visitation sequences in each foraging bout (raw data available in Lihoreau *et al*. [23]; electronic supplementary material, table S4). The following variables were calculated:

— Length of the flower visitation sequence: number of flower visits during a foraging bout.
— Flight duration (s): latency time between two successive visits to a different or the same flower.
— Probability of visiting a given flower during a given foraging bout.
— Probability of revisiting the same flower during a foraging bout, including consecutive revisits to the same flower.
— Number of different flowers: number of different flowers visited during a foraging bout (from 1 to 5).
— Cumulative number of distinct flowers that the bee encountered since the beginning of the experiment (flowers found).
— Distance from nest (m): mean distance between each flower visited during a foraging bout (including revisits) and the nest. This was estimated taking into consideration that flower 1 was placed at 53.1 m, flower 2 at 105.7 m, flower 3 at 126.2 cm, flower 4 at 104.4 and flower 5 at 53.4 cm from nest. Thus, for an optimal trapline bout (minimizing overall travel distances) the mean distance from the nest during the bout is approximately 89 m ((53.1 + 105.7 + 126.2 + 104.4 + 53.4)/5).
— Trapline: the most common five-flower visitation sequence, excluding revisits, used by each bee [17].
— Probability of all 36 possible transitions between the five flowers, and between the flowers and the nest. Given the symmetrical distribution of the flowers, transitions were classified according to equivalent path information (see graphical representation in the electronic supplementary material, figure S20). For example, a transition from the nest to flower 1 would be equivalent to the transition from the nest to flower 5, given that both flowers are positioned at the same distance from the nest and at the same relative angle, but in opposite direction. We also estimated 3-, 4- and 5-flower transition probabilities. For example, 3-flower symmetrical transition type 1 contains the transition from the nest to flower 1 followed by flower 2 (N–1–2), and the transition from the nest to flower 5 followed by flower 4 (N–5–4). Four-flower symmetrical transition type 1 contains transitions N–1–2–3 and N–5–4–3, and type 2 the transitions 1–2–3–4 and 5–4–3–2. The 5-flower symmetrical transition considers transitions N–1–2–3–4 and N–5–4–3–2.
— Turning angles*:* angles estimated from *x-* and *y*-position coordinates of three successively visited flowers as the difference between the arrival direction and the departure direction at the middle flower [36]. Hence, 0° indicates a move straight ahead, and 180° a complete reversal in direction. Noteworthy, the five flowers were arranged so that choices of nearest neighbours were always consistent with choices of straightest movements (i.e. lowest turning angle). Mean turning angle was estimated as the mean of the absolute values of turning angles of a foraging bout.
— Shannon entropy (*H*): represents the uncertainty of a variable [48]. For a probability *p*(*x*), where *x* is a variable capturing the behavioural strategy of each bee, it is defined by
  4.1 $$(X)=-\sum p(x)\log_{2}p(x).$$

In our case we estimated *H*_trans_, where *p* was the probability of performing one of the 36 possible transitions between flowers and/or nest, and *H*_ft_, where *p* was the relative flight duration (i.e. flight duration/duration of a foraging bout).

— Determinism: metric for measuring sequence predictability by quantifying the number and length of recurrences and series of recurrences. In the case of trapline foraging, a recurrence occurs whenever a forager revisits a resource [32]. Thus, a recurrent series occurs when sequence elements are repeated in the same order (in either forward or reverse directions). Determinism is based on the proportion of recurrences (i.e. revisited behavioural sequences) that belong to a recurrent series of a minimum designated length. It thus represents the proportion of revisited flowers that were visited in the same continuous order in multiple parts of the visitation sequence. For a detailed description of the method applied to trapline foraging, refer to Ayers *et al*. [32]. In order to assess recurrence of sequences in consecutive bouts (indicative of memory acquisition), we estimated determinism every three consecutive bouts, and a minimum designated length of three flowers was used.

### 4.3. Unsupervised behavioural mapping

We built our input dataset with 11 behavioural variables, namely: (1) the length of the flower visitation sequence, (2) the per cent of immediate revisits to a flower, (3, 4, 5) the per cent of symmetrical 2-flower transitions (types 3, 4 and 5; electronic supplementary material, figure S20A), (6,7) the per cent of both types of 4-flower transition, (8) the per cent of 5-flower transition, (9) the determinism index (a metric for detection of recurrent patterns in a behavioural sequence [36]), (10) the number of flowers found and (11) the probability of visiting flower 3 (the farthest flower from the colony nest, figure 1).

We made an unsupervised mapping of the input dataset by means of the three step mapping protocol described in Garriga & Bartumeus [30], publicly available using the bigMap R-package [49] and explained in the electronic supplementary material, §1. This mapping protocol is mainly governed by a parameter called ‘perplexity’ comparable with the number of nearest neighbours that is employed in many manifold learners. To ensure the robustness of the results, we tested different values of perplexity (i.e. 30, 60, 90 with respect to a dataset size of *N* = 193). For each test, we performed three different runs. For each run, we checked all possible sets of input features from 2 (i.e. the first two principal components) to 11 (i.e. the whole set of principal components). After a thorough analysis of all the runs we identified a robust classification of the foraging bouts into three clusters with a clear semantics, namely NNV, Route Development and Traplining. A detailed explanation of the procedure, including variable and parameter selection is provided in electronic supplementary material, S1. To highlight the biological significance and robustness of the behavioural strategies found in our study, we compared our results with another dimensionality reduction method (i.e. non-negative matrix factorization) discussed in the electronic supplementary material, S2.

### 4.4. Statistical analyses

We used the Kruskal and Wallis one-way analysis of variance by ranks to assess differences in foraging bout variables between behavioural strategies. We performed pairwise comparisons using Tukey and Kramer (Nemenyi) test with Tukey-Dist approximation for independent samples test, using the functions kruskal.test and posthoc.kruskal.nemenyi.test in the PMCMR R-package [50]. For graphical representations, we showed boxplots with median and 25% and 75% quantiles.

## Supplementary Material

Bumblebees learn foraging routes through exploitation-exploration cycles

## Data accessibility

This article has no additional data.
