# Supplementary material for: Bumblebees learn foraging routes through exploitation–exploration cycles
Source: J R Soc Interface. 2019 Jul 10;16(156):20190103. doi: 10.1098/rsif.2019.0103 (PMC6685008; doi:10.1098/rsif.2019.0103)
Supplement: Bumblebees learn foraging routes through exploitation-exploration cycles [file rsif20190103supp1.docx]

**Bumblebees learn foraging routes through exploitation-exploration cycles**

**Jackelyn M. Kembro**^1,2,3^**, Mathieu Lihoreau**^4^**, Joan Garriga**^3^**, Ernesto P. Raposo**^5^**, Frederic Bartumeus**^3,6,7,*^

^1^ Universidad Nacional de Córdoba Facultad de Ciencias Exactas, Físicas y Naturales, Instituto de Ciencia y Tecnología de los Alimentos and Cátedra de Química Biológica.

^2^ Concejo de Invesigaciones Cientificas y Tecnologicas. Instituto de Investigaciones Biológicas y Tecnológicas.

^3^ Centre d’Estudis Avançats de Blanes (CEAB-CSIC).

^4^ Research Center on Animal Cognition (CRCA), Center for Integrative Biology (CBI); CNRS, University Paul Sabatier.

^5^ Laboratório de Física Teórica e Computacional, Departamento de Física, Universidade Federal de Pernambuco.

^6^ Centre de Recerca en Ecologia i Aplicacions Forestals (CREAF).

^7^ Institut Català de Recerca i Estudis Avançats (ICREA).

**Corresponding author*: Centre d’Estudis Avançats de Blanes (CEAB-CSIC). Carrer Cala Sant Francesc, 14, 17300 Blanes, Girona, Spain. Telephone: +34 972 33 61 01. [fbartu@ceab.csic.es](mailto:fbartu@ceab.csic.es)

**Supplementary material**

**Section S1. Unsupervised Behavioural Mapping with t-SNE**

Current uses of machine learning in behavioural ecology often entail behavioural annotation and classification of large-structured (multivariate) data. In supervised classification methods (e.g. random forests, artificial neural networks, support vector machines) a predefined set of (labelled) behaviours is used to train classifiers that can then assign those labels to behavioural instances not included in the training set. Behavioral data may come from accelerometers (1) or other types of sensors (2), combined with behavioural annotations either on wild or captive animals in order to build up a training set. Contrastingly, non-supervised machine learning methods can describe behavioural variability and organization with as few a priori assumptions as possible. These methods, and in particular tSNE, have been used to describe the organizational principles of behaviour, for example, in model organisms such as Drosophila (3, 4).

In a context of unsupervised learning on large structured and multivariate data, non-linear dimensionality reduction techniques (as opposed to linear embedding of data like in Principal Component Analyses or multidimensional scaling) is more appealing because: (i) knowing the data structure at the local scale is fundamental as the input data is likely to be organized in a nonlinear manifold of much lower dimension; and (ii) when reduced to a human readable scale (i.e.2 o 3 first components) linear techniques might be dropping off a significant amount of information that might be crucial for visualization and analysis of data. Among nonlinear dimensionality reduction techniques t-SNE (5, 6) shows up as an outstanding embedding algorithm for the visualization of high-dimensional data in a human readable dimension space. The main driver of the embedding process is to preserve local pairwise similarities. That is, local similarities in the input space are mapped as close distances in the embedded space but moderate or large dissimilarities are not specially preserved. The t-SNE achieves this by expressing the set of pairwise similarities into a joint probability distribution in both, the input (high dimensional) space and the embedded (low dimensional) space. Afterwards, t-SNE minimizes the divergence between the two distributions. Here, we have used the parallelized version of the t-Stochastic Neighbouring Embedding algorithm (t-SNE) (4,5,7) developed in Garriga and Bartumeus 2018 (9) and presented as an R-package (bdMap_2.0.0).

A key aspect of any unsupervised mapping method (7) is the selection of input variables and the pre-processing of the data. In our case, out of approximately 150 possible variables estimated from the field data (available at Lihoreau et al. 2012 (8)), we preselected 11 variables (Table 1 in the main MS) based on minimum correlation between variables, high biological significance, and those that compress maximum information. For example, “probability of transition” was preferred over “number of transitions”, and instead of using the 36 possible probabilities of transition between flowers/nest, we used “probability of symmetrical transitions”. Interestingly, working out the flower transitions information in a very different way, i.e. using Nonnegative Matrix Factorization (S2 Supplementary Information), we can see correspondences between different types of flower transition information and the tSNE clusters obtained, emphasizing the robustness of our results to different types of metrics or procedures.

The raw data has been preprocessed by performing a principal component analysis (PCA) and a posterior whitening (i.e., renormalization of principal components). This process yields a set of input data with a diagonal covariance matrix, i.e., all components are uncorrelated and with variance 1. Afterwards, all pair-wise distances are computed and transformed into similarities based on a given value of perplexity. The perplexity is a key parameter of tSNE which defines a neighbourhood region of data points over which similarities will be computed (1,5). The essence of the tSNE procedure is transforming local similarities into a probability distribution of picking each datapoint as one of its neighbours, defined by the perplexity parameter. In this particular case, the “neighbouring” perplexity value (ppx=60) was selected to be roughly as N/3 (where N is the datasize) as this gave us a compromise between the global structure and local data variability (9). The full tSNE and mapping protocol parallelized parameterization (see Garriga and Bartumeus 2018 (9), and bigMap package) was set to be: ppx=60, pkde=30, rounds=8, layers=19,threads=20.

Herein, we evaluated the relative importance of each of the preselected variables and assessed the number of PCA dimensions that should be used in the tSNE analysis. As described in detail below, from these tests we selected the first 5 PCA dimensions, and kept all the 11 variables for tSNE analysis, considering that (i) all variables contributed relevantly to each axis, and (ii) this number of dimensions provide the most consistent and robust tSNEs. The resulting tSNE is observed in Figure S1 (see also another version of this Figure in Fig.1).


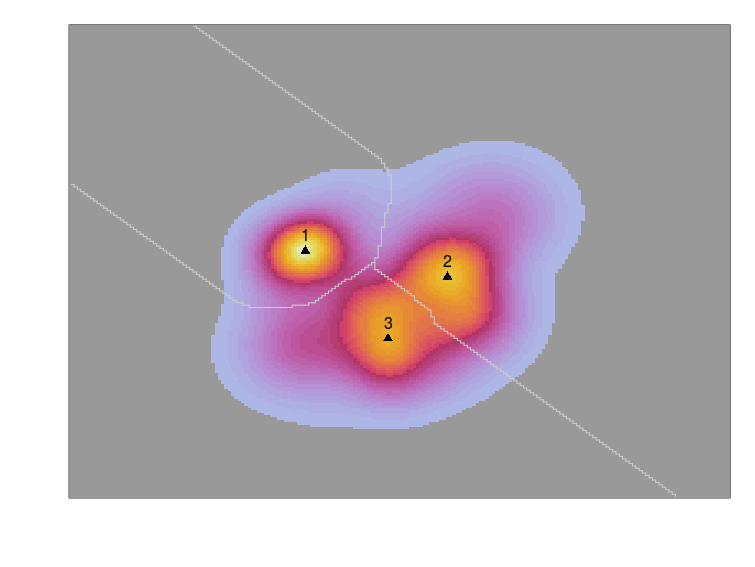


**Fig. S1.** Resulting t-SNE analysis with 3 clusters obtained using all 11 input variables and the first 5 dimensions of the Principal Component Analysis.

First, we evaluated the relative weights of each input variable in each of the PCA dimensions (S0 Table). Clearly, the 11 variables contribute relevantly (coeficients above 0,20, for example) along the different dimensions, from 1 to 11. Thus, making it hard to eliminate variables, and suggesting the importance of maintaing all the full set of 11 variables.

**Table S0.** Relative weights of each of the eleven input variables in each of the Principal Components (PC) dimensions.

|  | **Length** | **ImRev** | **pSym3** | **pSym4** | **pSym5** | **pSym4fa** | **pSym4fb** | **pSym5f** | **Det** | **nFlower** | **pFlower3** |
| --- | --- | --- | --- | --- | --- | --- | --- | --- | --- | --- | --- |
| **PC1** | -0.89 | -0.01 | -0.01 | -0.01 | -0.01 | -0.01 | -0.01 | 0.00 | -0.04 | -0.45 | -0.01 |
| **PC2** | -0.46 | -0.01 | 0.02 | 0.02 | 0.01 | 0.02 | 0.02 | 0.01 | 0.10 | 0.88 | 0.02 |
| **PC3** | -0.01 | 0.19 | -0.12 | -0.14 | -0.16 | -0.24 | -0.25 | -0.23 | -0.85 | 0.11 | 0.00 |
| **PC4** | -0.01 | 0.51 | -0.26 | -0.27 | -0.07 | -0.35 | -0.32 | -0.30 | 0.48 | -0.02 | -0.21 |
| **PC5** | 0.01 | -0.49 | -0.38 | -0.20 | 0.34 | -0.07 | 0.01 | 0.02 | -0.07 | 0.03 | -0.67 |
| **PC6** | -0.01 | 0.66 | -0.13 | -0.02 | 0.20 | 0.33 | 0.35 | 0.39 | -0.17 | 0.01 | -0.30 |
| **PC7** | 0.00 | 0.09 | -0.25 | 0.34 | 0.68 | -0.41 | 0.17 | -0.10 | -0.04 | -0.01 | 0.37 |
| **PC8** | 0.00 | 0.00 | 0.49 | -0.75 | 0.35 | -0.17 | 0.13 | 0.09 | -0.03 | 0.00 | 0.14 |
| **PC9** | 0.00 | 0.11 | 0.49 | 0.27 | 0.42 | 0.33 | -0.39 | -0.40 | -0.03 | 0.00 | -0.28 |
| **PC10** | 0.00 | 0.05 | 0.41 | 0.27 | -0.23 | -0.50 | 0.51 | -0.17 | -0.01 | 0.00 | -0.40 |
| **PC11** | 0.00 | -0.02 | -0.21 | -0.22 | -0.03 | 0.38 | 0.50 | -0.71 | 0.01 | 0.00 | 0.12 |

*Length*: the length of the flower visitation sequence. *ImRev*: the probability of immediate revisits to a flower. pSym4-5: the probability of symmetrical 2-flower transitions types 3, 4, and 5 (Fig. S8C, D, and E, respectively). *pSym4fa* and *pSym4fb*: the probability of 4-flower transition types 1 and 2, respectively (Fig. SJ, K). *pSym5f*: the probability of 5-flower transition (Fig. S8L, respectively). *Det*: the determinism index, *nFlower*: the numbers of different flowers visited, *pFlower3***:** the probability of visiting flower 3.

Second, we assesed the number of dimensions of the PCA that should be selected in the tSNE analyis. Different tSNE landscapes were obtained as we added PCA dimensions one by one, from 2 to 11 (Fig. S2). Except for the case of dimension 2, groups of 3 or 4 clusters are quite consistent as we increase PCA dimensions in the tSNE, however, the cluster #4 looks not very representative in terms of points density, and could be considered secondary. Note that from dimensions 5-8 three stable groups emerge. This is better illustrated in Figure S3 which summarizes the variation in the number of unsupervised clusters obtained with our mapping protocol as we increase the number of input PCA dimensions. Of notice is the stability of the results when incorporating 5,6, or 7 dimensions. In this regime, a good compromise between too few dimensions and a lot of dimensions is found. Although the addition of 9 or 11 dimensions also seem to stabilize the clustering, following the Occam’s Razor law of parsimony (10) we chosed the lowest number of dimensions that presents a stable output regarding the number of clusters.


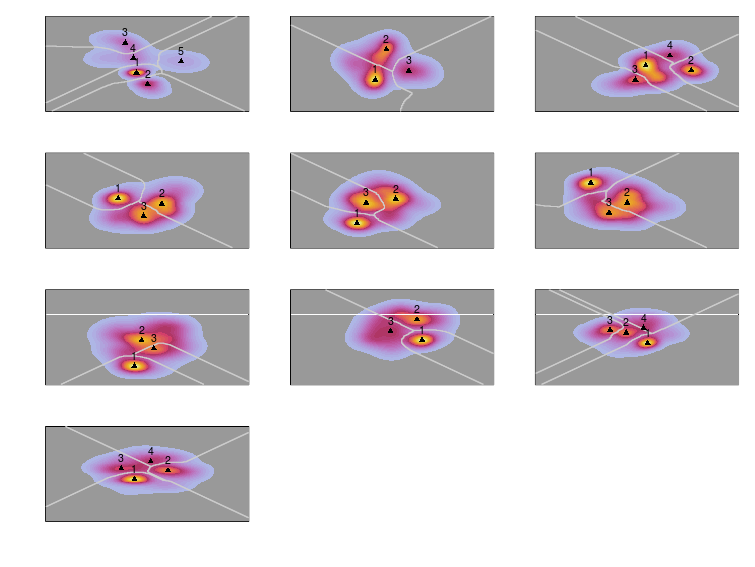


2D 3D 4D

5D 6D 7D

8D 9D 10D

11D

**Fig. S2.** The tSNE landscapes and unsupervised clustering obtained when using an increasing number of input dimensions of the Principal Component Analysis from 2 to 11 dimensions (2D-11D).


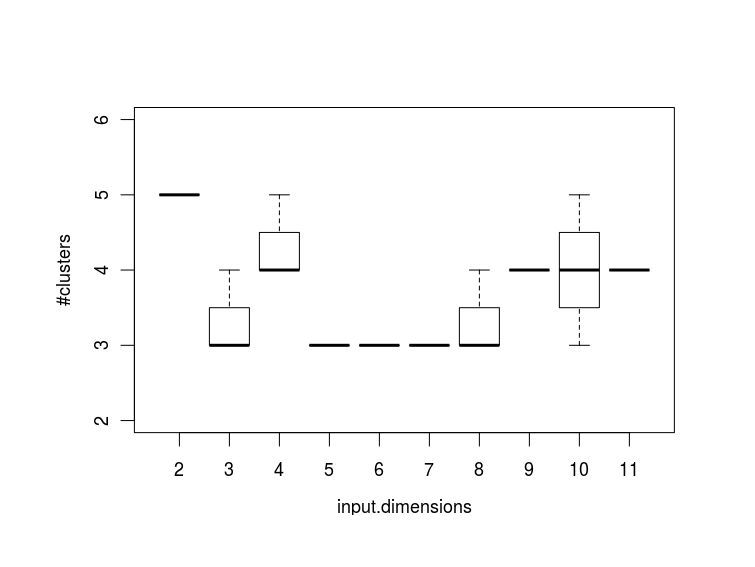


**Fig. S3.** Blox plot of the number of clusters obtained with tSNE and clustering protocol when using different number of input Principal Component dimensions. Three 3 tSNE runs were performed per dimension.

A separate complementary approach is to look at the landscape properties themselves, in particular, its embeding cost and its robustness. The embedding cost is a measure of the loss of information when converting high-dimensional data into a low-dimensional (embedded) landscape. As we incorporate more dimensions, we should expect a lower cost function, as more information is added, and the embedding represents better the high-dimensional data. The decayment curve can tell us which dimensions helps us better to decrease the cost function, and whether some strong downward shifts may exist. The Figure S4 shows a smooth and slow decay. In particular, about dimensions 5-6 almost flat. At 9,10,11 dimensions the cost seems to be stabilized. Provided that the cost difference is rather small for the tSNE analysis with 5 compared to 11 dimensions we have mantained our Occam’s Razor criteria.


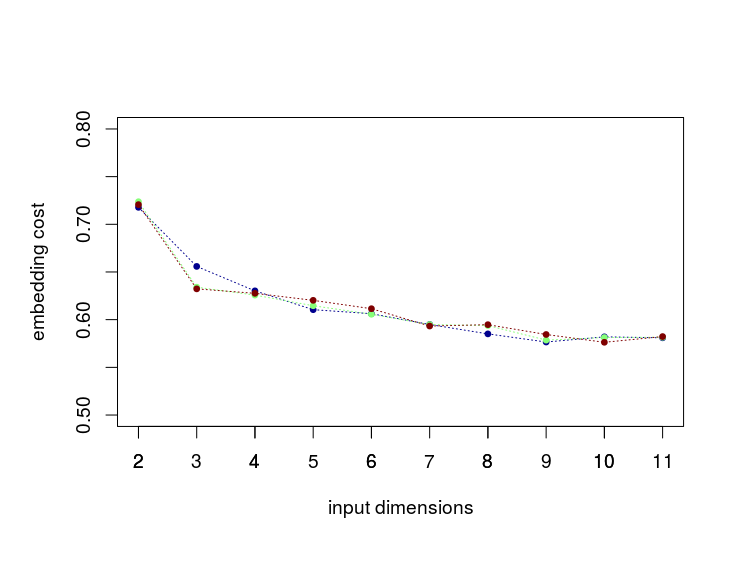


**Fig. S4.** Embedding cost of 3 tSNE runs as a function of the number of dimentions of the Principal Component Analysis used in the tSNE.

Finally, we also performed a tSNE landscape robustness analysis. We evaluated (i) the number of matches in the resulting number of clusters, and (ii) the consistency of our cluster labels evaluated on the basis of confusion matrices (average F-measure), for each PCA set of components (dimensionality), and out of 3 run tests (or replicates) per dimensionality. In Figure S5, we show the results for pair-wise comparisons between independent tSNE mapping protocols with the same set of parameters but incorporating 2, 3,…,11 PCA components as input features. Each cell in the matrix entails a two-dimensionality comparison (axis X and Y), that consist on 9 pair-wise comparisons obtained on the basis of three independent runs of the tSNE mapping protocol per dimensionality. We can observe a very stable (robust) ouput, with 3 final clusters, for comparisons involving from 5 to 7/8 dimensions (centre of the matrix in green).


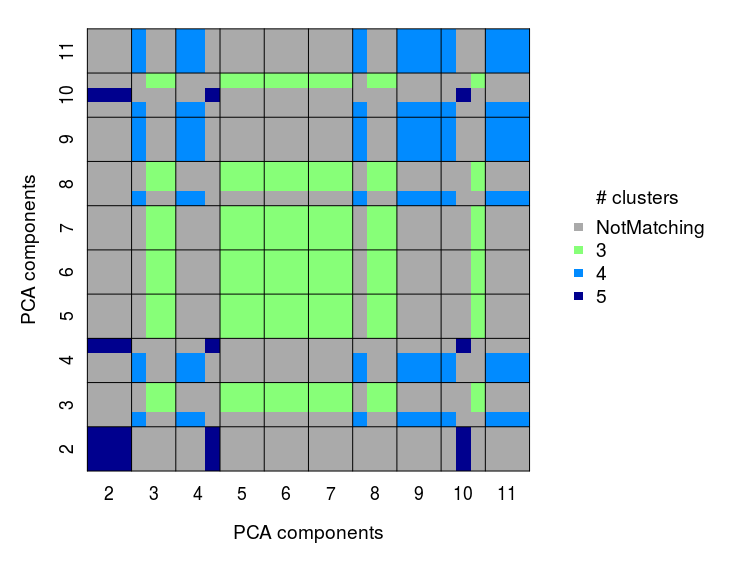


**Fig. S5.** Pair-wise comparisons of the number of clusters obtained from independent tSNE mapping protocols with different dimensionality. For each dimensionality (from 2 to 11 PCA components) we run 3 independent tSNE mapping protocols. In each cell, we show the results of 9 pair-wise comparisons between the 2 specified dimensionalities (number of PCA components). Colors show the resulting number of clusters. “Not matching” represents the cases were the number of clusters did not match. Parameterization of the tSNE was always the same, only differing in the number of dimensions.

In Figure S6, we applied the same protocol as in Figure S5 but instead of the number of clusters we measured differences based on confusion matrices, and using the F-measure as the global accuracy index. Different colors show different values of the F-measure. In grey, we depict run tests where the number of resulting clusters were not the same, so that confusion matrix calculations could not be done. Concordantly with Figure S5, the Figure S6 revealed a very stable (robust) ouput with high accuracies when comparing runs (F-measures above 0.80), involving from 5 to 7/8 dimensions and ultimately leading to the three cluster solution.


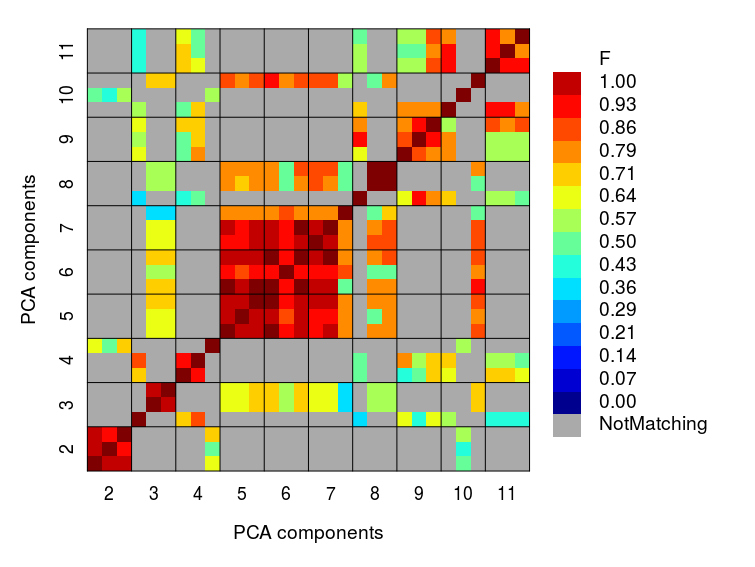


**Fig. S6.** Pair-wise comparisons of the accuracy (i.e., F-measure from a confusion matrix ) obtained from independent tSNE mapping protocols with different dimensionality. For each dimensionality (from 2 to 11 PCA components) we run 3 independent tSNE mapping protocols. In each cell, we show the results of 9 pair-wise comparisons between the 2 specified dimensionalities (number of PCA components). Colors show F-measure value. “Not matching” represents the cases were the number of clusters did not match, hence no confusion matrix could be computed. Parameterization of the tSNE was always the same, only differing in the number of dimensions.

Based on these analyses, we decided to choose one of the tSNE landscapes which included 5 PCA components or dimensions, which we believe are the most stable landscapes represented with the minimum amount of information necessary (in terms of PCA components), but which necessarily include the 11 variables initially chosen, as all of them contribute to build up the behavioural landscape and the final clustering.

Finally, we performed another simple test to evaluate the robustness of the clustering protocol used (9) which lead to discriminate 3 main dominant clusters. The test consisted in performing a partition with k-means (assuming k=3). In the latter, we inform the clustering method of the existence of three clusters, whereas in the former the clusters emerge without prior assumptions. We basically obtained identical results (Fig. S7): the three clusters were exactly the same, except for 6 mislabelled datapoints (out of 196). The relevant point, however, is that the assumption of a 3 cluster partition as being optimal (k-means approach) is only justified after all the tSNE data processing shown above.

**Fig. S7.** Partitioning the t-Stochastic Neighbouring Embedding (t-SNE) landscape into 3 dominant behavioural clusters, using the tSNE clustering protocol and k-means. Comparison of the tSNE clustering protocol which uses an adaptive kernel density and a watershed transformation (WSHD) with K-means (k=3) clustering algorithm. We obtained equivalent domains with both clustering methods, as indicated with black, red and green open circles. This comparison illustrates the robustness of our cluster partitioning in Fig. 1.

**References**

1. Nathan R, Spiegel O, Fortmann-Roe S, Harel R, Wikelski M, Getz WM. Using tri-axial acceleration data to identify behavioral modes of free-ranging animals: general concepts and tools illustrated for griffon vultures. J Exp Biol. 2012;215(Pt 6):986-96.
2. Carroll G, Slip D, Jonsen I, Harcourt R. Supervised accelerometry analysis can identify prey capture by penguins at sea. J Exp Biol. 2014;217(Pt 24):4295-302.
3. Berman GJ, Bialek W, Shaevitz JW. Predictability and hierarchy in Drosophila behavior. Proc Natl Acad Sci U S A. 2016;113(42):11943-8.
4. Berman GJ, Choi DM, Bialek W, Shaevitz JW. Mapping the stereotyped behaviour of freely moving fruit flies. J R Soc Interface. 2014;11(99).
5. van der Maaten LJP, Hinton GE. Visualizing high-dimensional data using t-sne. J Mach Learn Res. 2008;9:2579-605.
6. van der Maaten LJP, Postma E, Van den Herik J. Dimensionality reduction: a comparative review. J Mach Learn Res. 2009;10:66-71.
7. Todd JG, Kain JS, de Bivort BL. Systematic exploration of unsupervised methods for mapping behavior. Phys Biol. 2017;14(1):015002.
8. Lihoreau M, Raine NE, Reynolds AM, Stelzer RJ, Lim KS, Smith AD, et al. Radar tracking and motion-sensitive cameras on flowers reveal the development of pollinator multi-destination routes over large spatial scales. PLoS Biol. 2012;10(9):e1001392.
9. Garriga J, Bartumeus F. bigMap: Big Data Mapping with parallelized t-SNE. Journal of Statistical Software (submitted). J Stat Softw. 2018;submitted. Available at: https://arxiv.org/abs/1812.09869
10. Gauch HG. Scientific Method in Practice. Cambridge: Cambridge University Press; 2003.

**Section S2. Nonnegative matrix factorization (NNMF)**

NNMF (1-3) was performed on the 12 types of symmetrical transitions estimated (Fig. S8). Basically, NNMF is similar to PCA however it supposes that the input matrix (R) is of nonnegative values, where

V≈ W H

V is a matrix of size *n x m*, in our case, *n*-dimensional vector of symmetrical transitions (n=12), *m* being the total amount of bouts analyzed (m=193) and W the matrix of weights, of size n x r, H is the matrix of features of size r x m, and being r the number of r-dimensional features used.


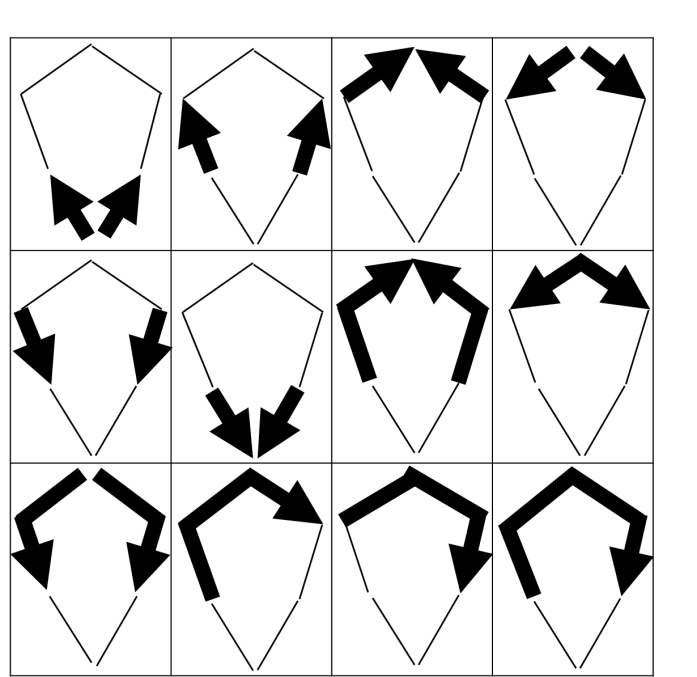


A B C D

E F G H

I J K L

**Fig. S8.** Scheme of the 12 types of symmetrical transitions used in the NNMF analysis. The first 6 (A-F) corresponded to two flower symmetrical transitions, (G-I) represent 3 flower symmetrical transitions, (J-K) are 4 flower symmetrical traditions and (L) shows a 5 flower symmetrical transition. In order to improve visualization, in the last 3 panels (J-L) only the transitions in one of the two directions is represented

NMF was completed using a number of features, r=7, that corresponded to a drop in residuals (1). The first 3 dimensions of the 7-dimentional features matrix ARE represented in Figure S9 (left), and dimension 1,3 and 4 are shown in Figure S9 (right). In this Figure, the colors (black, red and green) are a reference to the three behavioural strategies determined by the tSNE mapping protocol, namely Near-Nest Visits (NNV), Route Development, and Traplining, respectively.

**Fig. S9** **.** Three dimensional representation of the features matrix obtained with del NNMF. Colors represent the 3 behavioural strategies described by the tSNE mapping protocol, namely, nearest-nest exploitation (NNV, black), red (Route Development) and green (Traplining). The first 3 features (1, 2, 3) of the NNMF analysis are represented in the left panel, while features 1, 3, and 4 are shown in the right panel.


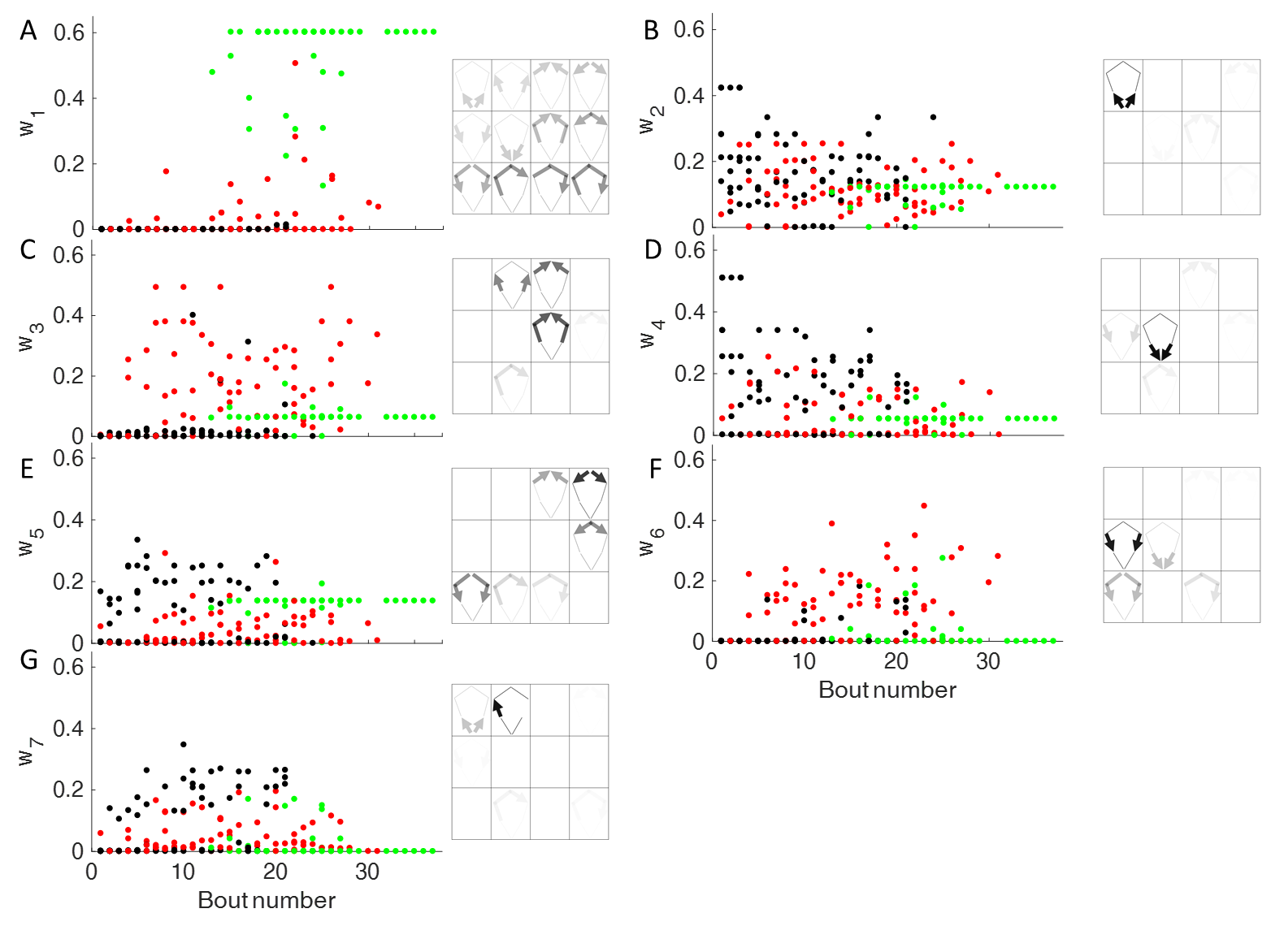


**Fig. S10.** Weights estimated for each of the 7 dimensions (W_1_-W_7_) of the features matrix result of NNMF as a function of the bout number. For comparative purpose, colors show the behavioural strategies obtained from the tSNE mapping protocol, namely nearest-nest exploitation (NNV, black), Route Development (red) and Traplining (green). The insets next to each panel show the relative contribution of each type of symmetrical transition (see Fig. S8 for description) to each of the features (W_1_-W_7_). Weights are represented using a greyscale, where black (≈1) indicates a high contribution and white (≈0) indicates no contribution.

The Figure S10 shows the weight of each of the 7 features represented as a function of the bout number. Next to each panel the relative contribution of each type of symmetrical transition to each feature is represented using a greyscale (black ≈1, white ≈0). Noticeably, the first feature (Fig. S10A) discriminates Traplining bouts, with important contributions of symmetrically transitions involving more than 2 flowers, and those directed towards the flower number 3. The third feature (Fig. S10C) discriminate the Route Development bouts, more strongly associated with two flower symmetrical transitions towards the second line of flowers and flower 3. Finally, NNV show predominant weights in the fourth feature (Fig. S10D), which captures the variation associated to returns to the nest. The mapping of the tSNE output with the NNMF axes (two radically different dimensionality reduction methods), highlight the biological significance and robustness of the behavioural strategies found in our study.

**References**

1. Desrochers TM, Jin DZ, Goodman ND, Graybiel AM. Optimal habits can develop spontaneously through sensitivity to local cost. Proc Natl Acad Sci U S A. 2010;107(47):20512-7.
2. Lee DD, Seung HS. Learning the parts of objects by non-negative matrix factorization. Nature. 1999;401(6755):788-91
3. Lee DD, Seung HS. Algorithms for Non-negative Matrix Factorization. Adv Neural Inform Process Syst. 2001;13.

**Section 3. *Exploratory behaviour promotes multi-scale movement and enhanced spreading***

**Flight trajectories.**

Six individuals were tracked with harmonic radar to record their 2D flight trajectories as they searched for flowers and developed a route (Fig. S11). The radar was used to record 4 foraging bouts by a naïve bumblebee (i.e., an individual that had not been trained previously in the pentagonal array) and 12 foraging bouts by experienced bumblebees (i.e., 5 out of the 7 individuals that had been previously trained in the pentagonal array). Coordinates of the bumblebees were recorded every 3 s with a spatial resolution of ± 2–3 m (1), from which we calculated the speed distribution, flight speed autocorrelation, and mean square displacement (MSD) flown after departing from a flower until a different flower was reached, or in the case of immediate revisits the maximum distance from flower was reached. Average cruising velocity was estimated based on the probability density function of flight speed computed from the radar data (Fig. S12).


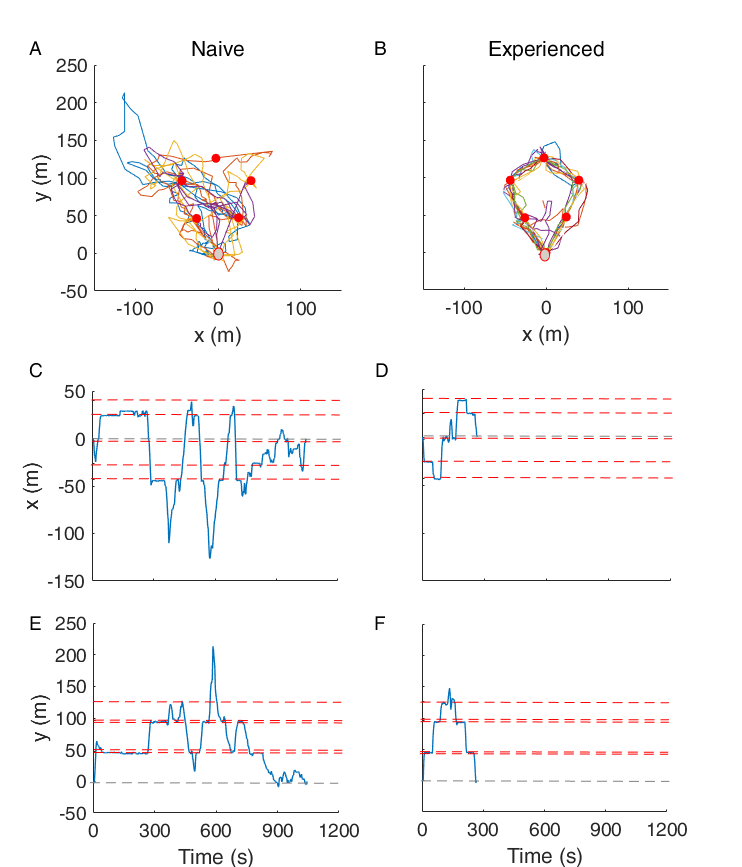


**Fig. S11. Radar trajectories of naïve and experienced bumblebees**. Complete recording of ﬂight patterns of A) 4 consecutive foraging bouts from a naive bumblebee and B) twelve foraging bouts from five experienced bumblebees in the pentagonal array of flowers (red filled circles). The looping characteristics observed are compatible with fast simulated annealing. Naive bumblebees venture further from flowers during flights, as reflected by a larger mean square displacement (Figs. S13C *vs.* S13D). An example of time courses of the x- and y-position coordinates in a naive (C,E) and an experienced bumblebee (D,F), corresponding to the blue trajectory in panel A and B, respectively. The temporal dynamics are represented together with the x- or y-position coordinates of five ﬂowers (red dashed-lines) and the nest (grey dashed-line). Note that the naive bumblebee (A,C,E) tends to (i) overshoot flower positions (compared to experienced bumblebees, panels B, D, F), and (ii) to increase loop size around flowers through time (e.g., x-y temporal dynamics associated with the flower in position x=-50,y=+100, panels C,E) before returning to the nest (time spans from 800 to 1000 s in panels C,E).


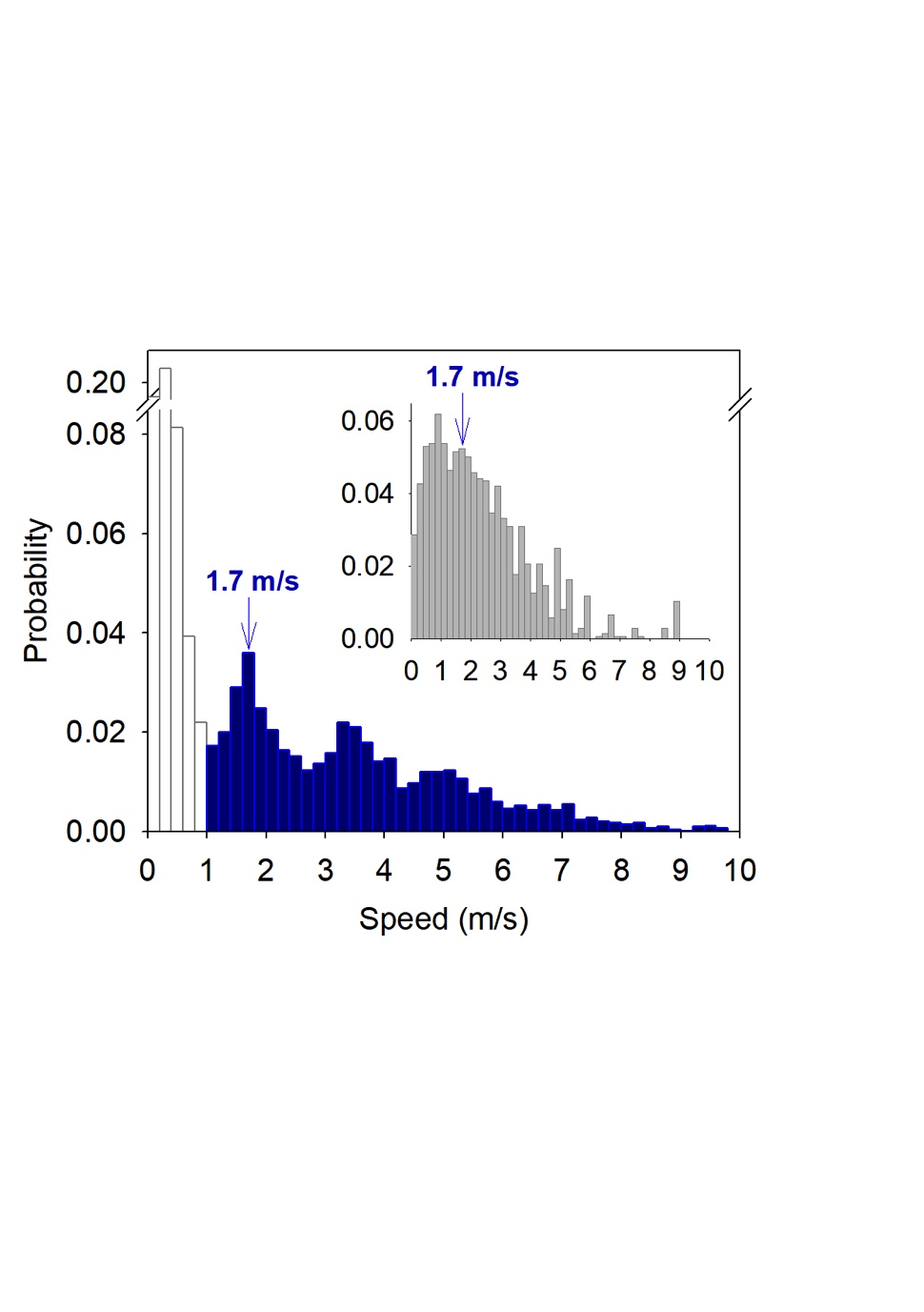


B

A

**Fig. S12. Flight speed and autocorrelations.** A) Probability density function of flight speed estimated from the radar data. White bars indicate speeds within the limit of sensibility of the radar to detect values different from zero. Inset shows flight speeds estimated as the distance between two successive flowers visits divided by the flight time. The most frequent speed distinguishable from zero was 1.7 m/s, as indicated by the blue arrow. This value is consistent with previous reports (Woodgate *et al.* 2017). B) The autocorrelation function was estimated for flight velocity separately for naive (open circles) and experienced (grey squares) bees during transitions between two flowers, or up to the maximum distance before starting a return trip to the same flower. Circles/squares and error bars represent mean and SEM, respectively. Correlations rapidly fall close to zero for time lags equal or larger than 6 s in both naive and experienced bumblebees.

**Characterizing flight time probability distributions**

In this section we provide the mathematical expressions and developments for the models analysed in the manuscript. We start with the general formalism that can be applied to any probability density function (pdf) $p(t)$ of flight times $t$, and then particularize in the following to the worked examples.

Consider initially that the flight times present lower and upper bounds, i.e., $t_{min}\leq t\leq t_{max}.$ The normalization condition in this case reads

$$\int_{t_{min}}^{t_{max}} p\left( t \right)dt=1.$$

The associated cumulated density function (cdf) $P(t)$ is found from $p(t)$ according to

$$P\left( t \right)=\int_{t_{min}}^{t} p\left( t' \right)dt'.$$

We comment that a random number generator can be obtained from the cdf by the standard inversion procedure, in which a random number $u$ uniformly drawn in the interval $0\leq u\leq1$ is assigned so that $u=P(t)$, and the resulting equation for $t$ is solved either analytically (if possible) or numerically. At the end, the obtained values of $t$ will be distributed according to the pdf $p\left( t \right).$

It is also useful to provide expressions for the analysis of pre-binned data. In this case, we initially define the following quantities,

$t_{j-1}=t_{1}+(j-2)\omega$,

for integer *j* > 1, and

$$t_{j}=t_{1}+(j-1)\omega,$$

where $\omega$ is the small length of the interval between two consecutive discrete values of $t_{j}$, i.e.,

$$t_{j}=t_{j-1}+\omega.$$

In the specific condition with a lower bound, we generally choose $t_{1}{=t}_{min}.$ We also define the probability of a given flight time to be in bin $j$ as

$$P\left( j|\omega\right)=\int_{t_{j-1}}^{t_{j}} p\left( t \right)dt.$$

Therefore, by using the definition above of the cdf, we obtain

$${P\left( j|\omega\right)=P\left( t_{j} \right)-P(t}_{j-1}).$$

In the following, the above general expressions are applied to the specific flight time models analysed in the present work.

*3.1 Streched exponential (str Exp)*

We consider the pdf of the stretched exponential model to be defined as

$$p\left( t \right)=A\exp\left( -{(t/\theta)}^{\beta} \right),$$

for $t\geq t_{min},$ $\theta>0$ and $\beta>0.$ Notice that this pdf is not limited from above, i.e., $t_{max}\to\infty.$ Moreover, $p\left( t \right)=0$ if $t<t_{min}$. The normalization constant can be found by changing the variable $y={(t/\theta)}^{\beta}$, yielding

$$A=\frac{\beta}{\theta\Gamma\left( 1/\beta,{(t_{min}/\theta)}^{\beta} \right)},$$

where the upper incomplete gamma function reads

$$\Gamma\left( s,x \right)=\int_{x}^{\infty} z^{s-1}e^{-z}dz.$$

The same change of variables also allows to calculate the associated cdf as

$$P(t)=\frac{A\theta}{\beta}\left[ \Gamma\left( 1/\beta,{(t_{min}/\theta)}^{\beta} \right)-\Gamma\left( 1/\beta,{(t/\theta)}^{\beta} \right) \right],$$

for $t\geq t_{min},$ and $P\left( t \right)=0$ otherwise. Finally, by defining

$$y_{j}=\left( \frac{t_{j}}{\theta} \right)^{\beta},$$

we end up with

$$P\left( j|\omega\right)=\frac{A\theta}{\beta}\left[ \Gamma\left( 1/\beta,{(y_{j-1}/\theta)}^{\beta} \right)-\Gamma\left( 1/\beta,{(y_{j}/\theta)}^{\beta} \right) \right],$$

if $t_{j-1}\geq t_{min},$ and $P\left( j|\omega\right)=0$ otherwise.

*3.2 Double and Triple Exponentials* *(double and triple Exp)*

The general pdf of the hyper-exponential distribution is given by

$$p\left( t \right)=A\sum_{i=1}^{N} \frac{B_{i}}{\tau_{i}}\exp\left( -t/\tau_{i} \right),$$

for $t_{min}\leq t\leq t_{max},$ and $p\left( t \right)=0$ otherwise, where $N$ is the number of exponential components ($N=2$ for the double exponential and $N=3$ for the triple one, and so on), $\tau_{i}>0$ denote the time decay constants$,$and the statistical weights of the exponential functions are such that

$$\sum_{i=1}^{N} B_{i}=1.$$

The normalization constant is given by

$$A=\frac{1}{\sum_{i=1}^{N} B_{i}\exp\left( -t_{min}/\tau_{i} \right)-\sum_{i=1}^{N} B_{i}\exp\left( -t_{max}/\tau_{i} \right)}.$$

The associated cdf thus reads

$$P(t)=A\sum_{i=1}^{N} B_{i}\exp\left( -t_{min}/\tau_{i} \right)-A\sum_{i=1}^{N} B_{i}\exp\left( -t/\tau_{i} \right),$$

for $t_{min}\leq t\leq t_{max},$ with $P\left( t \right)=0$ if ${t< t}_{min}$ and $P\left( t \right)=1$ if $t>t_{max}.$ A similar calculation leads to the pre-binned expression

$$P\left( j|\omega\right)=A\sum_{i=1}^{N} B_{i}\exp\left( -t_{j-1}/\tau_{i} \right)-A\sum_{i=1}^{N} B_{i}\exp\left( -t_{j}/\tau_{i} \right),$$

if $t_{min}\leq t_{j-1}\leq t_{max},$ and $P\left( j|\omega\right)=0$ otherwise.

*3.3 Composite function A* *(Exp+PL+strExp)*

We call the composite function A as the pdf that results from the combination of an exponential function for small time values, a power-law function for the intermediate and large values, and a stretched-exponential function as the upper tail regime (largest and very rare values):

$$p\left( t \right)= \left\{ \begin{aligned} Ae^{-\alpha t}, t_{1}\leq t<t_{2}, \\ Bt^{-\mu}, t_{2}\leq t<t_{3}, \\ Ce^{-{(t/\theta)}^{\beta}}, t\geq t_{3}. \end{aligned} \right.$$

We take $t_{1}{=t}_{min}$ as before. Now, since the pdf $p\left( t \right)$ is defined in partitioned intervals then we must assure that it is continuous and smooth at the border points $t{=t}_{2}$ and $t{=t}_{3}.$ This can be achieved by imposing the continuity of $p\left( t \right)$ and its derivative $dp/dt$ at these points. This implies the following set of equations:

$$A\exp\left( -{\alpha t}_{2} \right)=Bt_{2}^{-\mu},$$

$$\alpha A\exp\left( -{\alpha t}_{2} \right)=\mu Bt_{2}^{-\mu-1},$$

$$Bt_{3}^{-\mu}=C\exp\left( -{(t_{3}/\theta)}^{\beta} \right),$$

$$\mu Bt_{3}^{-\mu-1}=\frac{\beta Ct_{3}^{\beta-1}}{\theta^{\beta}}\exp\left( -{(t_{3}/\theta)}^{\beta} \right),$$

from those we find

$$\alpha=\frac{\mu}{t_{2}},$$

$$B{=At}_{2}^{\mu}e^{-\mu},$$

$$\theta=t_{3}\left( \frac{\beta}{\mu} \right)^{1/\beta},$$

$$C{=A\left( \frac{t_{2}}{t_{3}} \right)^{\mu}t}_{2}^{\mu}e^{-\mu(1-1/\beta)}.$$

Next, we calculate the normalization constant by changing the variable $y={(t/\theta)}^{\beta}$ in the normalization integral, leading to

$$A= \frac{\mu e^{\mu}}{t_{2}}\left\{ -1+e^{\mu\left( 1-t_{1}/t_{2} \right)}+\frac{\mu}{(1-\mu)}\left[ \left( \frac{t_{3}}{t_{2}} \right)^{1-\mu}-1 \right]+\left( \frac{t_{3}}{t_{2}} \right)^{1-\mu}\left( \frac{\beta}{\mu} \right)^{1/\beta-1}e^{\mu/\beta}\Gamma\left( 1/\beta,\mu/\beta\right) \right\}^{-1}.$$

Notice, therefore, that the coefficient $A$ is expressed above in terms of the pdf parameters $\mu$ and $\beta,$ besides the time boundary values $t_{1},t_{2},t_{3}.$ Further, by substituting this expression for $A$ into the above equations for $B$ and $C$, we obtain these coefficients also as functions of the same parameters.

The calculation of the associated cdf must be done for each interval separately. Therefore, in the first interval we find

$$P\left( t \right)=\frac{At_{2}}{\mu}\left( e^{-{\mu t}_{1}/t_{2}}-e^{-\mu t/t_{2}} \right),$$

for $t_{1}\leq t<t_{2},$ with $P\left( t \right)=0$ if ${t< t}_{1}.$ For the second interval, we obtain

$$P\left( t \right)\mathcal{=A+}\frac{B}{(1-\mu)}\left( t^{1-\mu}-t_{2}^{1-\mu} \right),$$

for $t_{2}\leq t<t_{3},$ where

$$\mathcal{A=}\frac{At_{2}}{\mu}\left( e^{-{\mu t}_{1}/t_{2}}-e^{-\mu} \right).$$

And the third interval is described by

$$P\left( t \right)\mathcal{=A+B+}C\frac{t_{3}}{\beta}\left( \frac{\beta}{\mu} \right)^{1/\beta}\left[ \Gamma\left( 1/\beta,\mu/\beta\right)-\Gamma\left( 1/\beta,(\mu/\beta){(t/t_{3})}^{\beta} \right) \right],$$

for $t\geq t_{3},$ where

$$\mathcal{B=}\frac{B}{(1-\mu)}\left( t_{3}^{1-\mu}-t_{2}^{1-\mu} \right).$$

Similarly, the pre-binned expressions are also defined for each range, with some care needed at the border separating each two adjacent intervals:

$$P\left( j|\omega\right)=\frac{At_{2}}{\mu}\left( e^{-{\mu t}_{j-1}/t_{2}}-e^{-\mu t_{j}/t_{2}} \right),$$

for $t_{1}\leq t_{j-1}<t_{2}-\omega,$ with $P\left( j|\omega\right)=0$ if ${t_{j-1}< t}_{1},$

$$P\left( j|\omega\right)=\frac{At_{2}}{\mu}\left( e^{-{\mu t}_{j-1}/t_{2}}-e^{-\mu t_{1}/t_{2}} \right)\mathcal{+ A+}\frac{B}{(1-\mu)}\left( t_{j}^{1-\mu}-t_{2}^{1-\mu} \right),$$

for $t_{2}-\omega\leq t_{j-1}<t_{2},$

$$P\left( j|\omega\right)=\frac{B}{(1-\mu)}\left( t_{j}^{1-\mu}-t_{j-1}^{1-\mu} \right),$$

for $t_{2}\leq t_{j-1}<t_{3}-\omega,$

$$P\left( j|\omega\right)=\frac{B}{(1-\mu)}\left( t_{2}^{1-\mu}-t_{j-1}^{1-\mu} \right)\mathcal{+B+}C\frac{t_{3}}{\beta}\left( \frac{\beta}{\mu} \right)^{1/\beta}\left[ \Gamma\left( 1/\beta,\mu/\beta\right)-\Gamma\left( 1/\beta,(\mu/\beta){(t_{j}/t_{3})}^{\beta} \right) \right],$$

for $t_{3}-\omega\leq t_{j-1}<t_{3},$ and, finally,

$$P\left( j|\omega\right)=C\frac{t_{3}}{\beta}\left( \frac{\beta}{\mu} \right)^{1/\beta}\left[ \Gamma\left( 1/\beta,(\mu/\beta){(t_{j-1}/t_{3})}^{\beta} \right)-\Gamma\left( 1/\beta,(\mu/\beta){(t_{j}/t_{3})}^{\beta} \right) \right],$$

for $t_{j-1}\geq t_{3}.$

*3.4 Composite function B* *(Exp+TPL)*

The last pdf investigated in this work is a composite function that combines an exponential function for small time values and a truncated power-law function for the intermediate and large values, the tail being represented by a sharp cut-off value, which was not considered in the previous composite pdf:

$$p\left( t \right)= \left\{ \begin{aligned} Ae^{-\alpha t}, t_{1}\leq t<t_{2}, \\ Bt^{-\mu}, t_{2}\leq t\leq t_{3}, \end{aligned} \right.$$

where $t_{1}{=t}_{min},$ $t_{3}{=t}_{max}$, and $p\left( t \right)=0$ if $t<t_{1}$ or $t>t_{3}$.

In a way similar to the calculation using the previous composite pdf, the normalization constant now reads

$$A= \left\{ e^{-\alpha t_{1}}-e^{-\alpha t_{2}}+\frac{t_{2}^{\mu}e^{-\mu}}{\mu}\left[ t_{2}^{1-\mu}-t_{3}^{1-\mu} \right] \right\}^{-1}.$$

The associated cdf has essentially the same expressions as for the previous composite function for the intervals $t_{1}\leq t<t_{2}$ and $t_{2}\leq t<t_{3},$ but with $A$ given above and $P\left( t \right)=1$ for $t>t_{3}.$ The same also happens to the pre-binned calculations.

**Maximum likelihood fitting procedure**

We fit the probabilistic models for flight times to empirical data by means of maximum-likelihood and Akaike Information Criteria model selection standard procedures (2, 3). Given that our data showed discrete temporal resolution (minimum observable value of 1 second), we used the pre-binned approach suggested in Edwards *et al.* 2007 (Supplementary Information), assuming a binwidth of 1 s.

We used the R-Package “bbmle” (Bolker and R Development Core Team, 2016) to build the probability/cumulative densities and likelihood functions. We used the “L-BFGS-B” optimization algorithm from the “stats” core-package (R Core Team 2017), which allows for *bounded* parameter space exploration.

**Results from radar records**

An in-depth look into the distributions of flight durations between flower visits, or simply flight times, (Fig. S13 and Tables S1 and S2) reveals the emergence of multiple spatiotemporal scales beyond normal (i.e. Gaussian or exponential) variability. A synthetic bumblebee at a cruising velocity of 1.7 m/s (see Material and Methods and Fig. S12A) and using the shortest route to visit all flowers in the experimental array (one time each), would lead flight durations of 29.4 s (50 m at 1.7 m/s) between nearest flowers. Assuming normal variation around this value (i.e. fluctuations in cruising speed or turning behaviour), traplining should lead to an exponential flight time distribution with characteristic scale Θ=29.4 s (Fig. S13A, dark grey line). Indeed, the best models for flight time distributions are double exponentials (Tables S1 and S2). In the Traplining strategy a dominant characteristic scale of 30 s appears (Fig. S13B, Table S2), while in the NNV strategy the dominant flight time scale becomes larger (60 s) and a subdominant but non-negligible second scale of about 400 s emerges (Fig. 13B, Table S1). In the Route Development strategy, the two-scales (about 40 s and 300 s) become more apparent and balanced (Fig. 12B, Table S1), so that the bi-exponential model becomes hard to discriminate from other models showing more than two characteristic scales or power-law regimes (Tables S1 and S2).

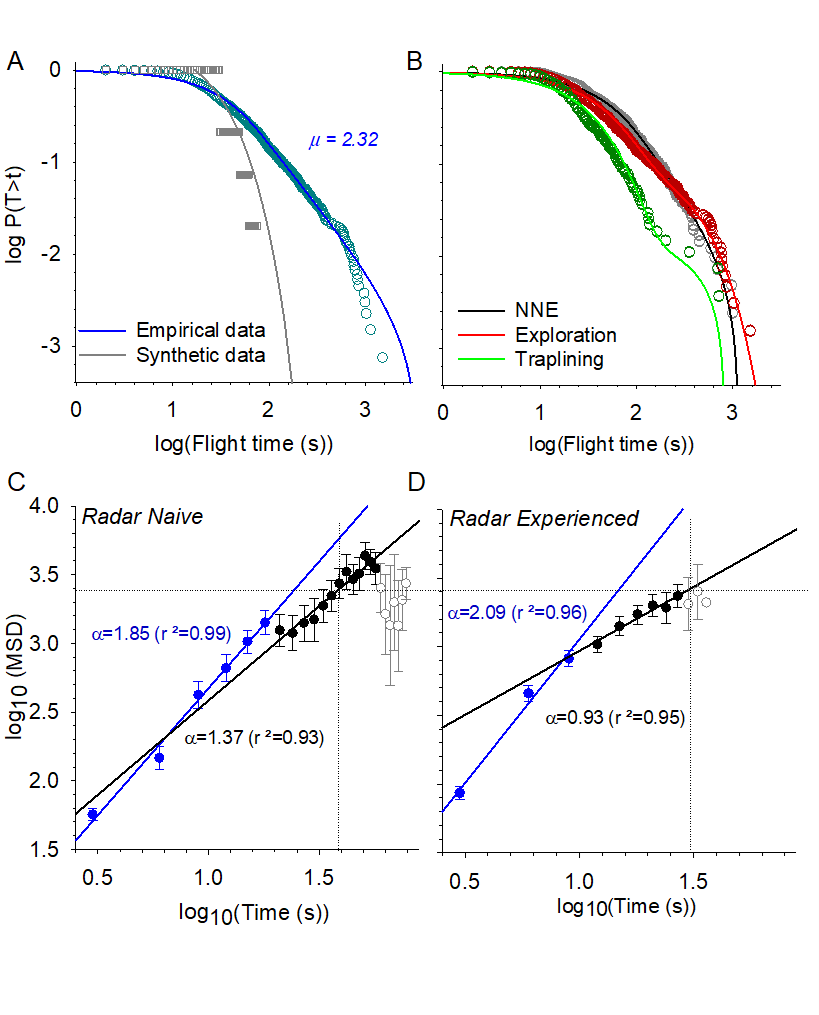


**Fig. S13. Cumulative distributions of flight durations between successive flower visits and flower departure spreading dynamics.**

Top panels: A) Cumulative probability density distributions of all 1336 empirical flight times (blue circles) obtained from all foraging bouts analysed and synthetic data (grey squares) representing the occurrence of flight duration (i.e., the duration of the flights between flowers) that would have been obtained if bees would have flown directly between flowers at a cruising velocity of about 1.7 m/s (see S18 Fig. for the estimation of speed). The dark grey line represents a stretched exponential pdf fit (with β=1 and characteristic time scale Θ=27.4 s). The blue line illustrates the behaviour of the composite pdf 2 fit (exponential + truncated power-law) of all flights. B) Cumulative probability density distributions of flight durations from foraging bouts of each of the three behavioural strategies: NNV (total 416 flights), Route Development (647 flights) and Traplining (273 flights). The black, red, and green lines illustrate the behaviour of the composite pdf 2 fit (exponential + truncated power-law) of those from NNV, Route Development and Traplining strategies, respectively. μ represents the maximum likelihood estimator for the slope of a truncated power law. See S2 Table for details on parameter estimation, confidence interval, log-likelihood and AIC. Bottom panels: Mean square displacement of naive (C) and experienced (D) bumblebees measured from each flower until reaching a new flower, or up to the maximum distance before starting a return trip to the same flower. Data taken from radar trajectories in Lihoreau et al. (4). Two distinct diffusion regimes (blue and black) are identified through regression lines (see main text for discussion). Horizontal dashed line marks the distance between nearest flowers in the experimental setup (50 m). Vertical dashed line is the time it takes an average bumblebee to depart 50 meters out of the current flower (naive: around 39 s; experienced: around 27 s).

**Table S1**. Likelihood comparison for different probabilistic models for flight times of all empirical data (ALL) and of each behavioural strategy: Near-Nest Visits (NNV), Route Development and Traplining. For the two selected models (Table S2), and whenever optimization procedures converged to a specific value, we provide the confidence intervals of the parameters in brackets [2.5%, 97.5%].

|  |  | All | NNV | Route Development | Traplining |
| --- | --- | --- | --- | --- | --- |
|  | N | 1334 | 411 | 652 | 271 |
|  | Minimum | 2 | 2 | 2 | 2 |
|  | Maximum | 3613 | 1145 | 3613 | 827 |
| *Str*  *exp* | Θ | 20.92 | 46.16 | 15.06 | 19.98 |
|  | Β | 0.60 | 0.76 | 0.53 | 0.711 |
| *double EXP* | P_1_ | 0.90  [0.87,0.92] | 0.95  [0.90, 0.98] | 0.87  [0.83 0.90] | 0.97  [0.94 0.99] |
|  | *P_2_ | 0.10  [0.08,0.13] |  |  |  |
|  | T_1_ | 42.64  [39.65,45.87] | 61.69  [54.46, 69.84] | 39.2  [35.24 43.70] | 31.09  [27.48, 35.29] |
|  | T_2_ | 289.79  [228.80,378.31] | 395.56  [169.40, NaN<+ | 289.08  [219.54 397.03] | 797.3  [205.51 NaN] |
| *triple EXP* | P_1_ | 0.90 | 0.95 | 0.87 | 0.97 |
|  | P_2_ | 0.001 | 0.001 | 0.001 | 0.0001 |
|  | *P_3_ | 0.099 |  |  |  |
|  | T_1_ | 42.64 | 61.68 | 39.21 | 31.08 |
|  | T_2_ | 55.07 | 71.68 | 55.28 | 0.0001 |
|  | T_3_ | 89.82 | 395.72 | 289.12 | 785.27 |
| *Exp+PL+*  *strExp* | Μ | 2.32 | 2.68 | 2.13 | 2.82 |
|  | Β | 0.52 | 1.01 | 0.48 | 1.01 |
|  | T_2_ | 98.04 | 161.30 | 83.97 | 82.34 |
|  | T_3_ | 999.70 | 865.83 | 999.77 | 826.85 |
| *Exp+TPL* | Μ | 2.32  [2.18,2.50] | 2.56 | 2.15 | 2.75 |
|  | T_2_ | 98.21  [87.47,111.98] | 151.84 | 84.75 | 80.06 |

**Table S2**. Model selection based on likelihood comparison for different probabilistic models for flight times of all empirical data (ALL) and of each behavioural strategy: Near-Nest Visits (NNV), Route Development and Traplining (ΔAIC=AIC_i_–AIC_min_). Selected models are shown in yellow. The double exponential pdf is selected for the data set NNV and Traplining, whereas two models are selected for Route Development, both the double exponential model and the composite pdf: exponential + truncated power law are both equally good (relative ΔAICc <2). Interestingly, when pooling all the data together the composite function~~:~~ exponential+ truncated power law is selected.

| ALL | PDF MODEL | ΔAICc | df | Akaike weigth |
| --- | --- | --- | --- | --- |
|  | *Exp+TPL* | 0 | 2 | 0.89 |
|  | *Exp+PL+strExp* | 4.2 | 4 | 0.11 |
|  | *doubleExp* | 26.8 | 3 | <0.001 |
|  | *tripleExp* | 30.8 | 5 | <0.001 |
|  | *strExp* | 184.4 | 2 | <0.001 |
| NNV | **PDF MODEL** | Δ**AICc** | **df** | **Akaike weigth** |
|  | *doubleExp* | 0 | 3 | 0.872 |
|  | *tripleExp* | 4.1 | 5 | 0.113 |
|  | *Exp+TPL* | 8.2 | 2 | 0.014 |
|  | *Exp+PL+strExp* | 14.0 | 4 | <0.001 |
|  | *strExp* | 41.2 | 2 | <0.001 |
| ROUTE DEVELOPMENT | **PDF MODEL** | Δ**AICc** | **df** | **Akaike weigth** |
|  | *doubleExp* | 0 | 3 | 0.559 |
|  | *Exp+TPL* | 1 | 2 | 0.336 |
|  | *tripleExp* | 4.1 | 5 | 0.073 |
|  | *Exp+PL+strExp* | 5.7 | 4 | 0.032 |
|  | *strExp* | 109.8 | 2 | <0.001 |
| TRAPLINING | **PDF MODEL** | Δ**AICc** | **df** | **Akaike weigth** |
|  | *doubleExp* | 0 | 3 | 0.886 |
|  | *tripleExp* | 4.1 | 5 | 0.111 |
|  | *Exp+TPL* | 12.1 | 2 | 0.0021 |
|  | *Exp+PL+strExp* | 16.7 | 4 | <0.001 |
|  | *strExp* | 60.8 | 2 | <0.001 |

The slower decay of flight durations in the Route Development strategy compared to the NNV and Traplining strategies (Fig. S13B) reflects a larger probability of long flights between flower visits, and some more heterogeneous behavioural dynamics, compared to those performed in NNV or Traplining bouts, concordantly with a larger H_fl_ (Fig. 2C). Such a broader continuum of medium-to-large flight durations (power law regime) becomes even more significant when pooling data of all foraging bouts and all bumblebees (Fig. S13A, Tables S1 and S2), that is, when the full mix of scales and behavioural strategies is considered. The model selected for the pooled dataset clearly shows a power law regime over a fixed range of scales (Fig. 11A, S3 Table), a signature mainly coming from the Route Development subset of foraging bouts. These results suggest that bumblebees perform flights much beyond the experimental scales most likely after fixing some initial route segments close to the nest, and when engaged in learning new routes further away.

**S3 Table.** Behavioural variables estimated from radar data of a naive bumblebee and 7 experienced bumblebees.

|  | Naive |  |  | Experienced |  | p-value |
| --- | --- | --- | --- | --- | --- | --- |
| Mean distance from nest (m) | 74 (68; 74) |  |  | 85 (75; 87) |  | 0.03 |
| Maximum distance from nest (m) | 161 (150; 163) |  |  | 130 (125; 138) |  | 0.001 |

Median (Q1; Q3). Statistically significant differences differ by p < 0.05 (Kruskal Wallis test).

Laboratory studies that have analysed bumblebee search flights suggest that naïve individuals spread out from visited flowers with a multi-scale looping pattern producing ever increasing back-and-forth movements from the focal point (5). These types of patterns resemble Fast Simulated Annealing (FSA, (6)), which has been suggested as a relatively simple yet highly efficient stochastic sampling strategy (5). Simulated Annealing involves a family of local search methods that can avoid local minima, different algorithms from this family spreading out from local minima at different rates. To further test this hypothesis in a much larger field setup, we analysed the spreading out from flowers (i.e., computing mean square displacements, MSD) based on radar-tracked motion of one naïve bumblebee (during its first four foraging bouts) and seven experienced bumblebees (during their last foraging bouts, bout number > 25) in the pentagonal array. The results suggest a complex spreading pattern with two diffusive regimes (Figs. S13C and S13D). In random walk processes, the MSD goes with time as $MSD\propto t^{-\alpha}$ , where $\alpha=1$ indicates normal diffusion, and $\alpha>1(\alpha<1)$ are indicative of super(sub)-diffusive regimes (34). Taking logarithms one can empirically estimate $\alpha$ by fitting a regression line and estimating the slope. The naïve individual departed from flowers showing enhanced diffusivity (7) for the first 18-20 s ($\alpha>1$; blue circles in Fig. S13C), then slowing down (black circles) as the likelihood to find a new flower increases. Experienced individuals departed from the flowers ballistically (for the first 10 s ($\alpha\approx2$; blue circles in Fig. S13D) to rapidly slow down to subdiffusive regime ($\alpha<1;$black circles) as they reach the next flower. The naïve individual clearly surpassed nearest inter-flower distances (dotted horizontal lines, 50 m) when departing from any flower in the array, whereas experienced foragers did not (white circles in Figs. S13C and D, and S11). The times to reach the first 50 m from the flower was approximately 39 s in the naïve bumblebee and 27 s in the experienced foragers (dotted vertical lines in Figs. S12C and 3D, respectively). The enhanced diffusivity shown by the naïve individual does not come from long-term velocity correlations, as both the naïve and the experienced bumblebees only show short-ranged velocity correlations of less than 6 seconds ( Fig. S12B). Instead, it comes from the increasingly large returning times to the focal flower as the bumblebee spread out (Fig. S11), which is consistent with a search process resembling FSA (5). On the contrary, experienced bumblebees showed a spreading pattern that is consistent with route following based on spatial memories, as the MSD dynamics (Fig. S13D) suggest a strong mapping of the spatiotemporal scales associated to the artificial flower array.

The radar data also showed that, for the first 4 foraging bouts, the naïve bumblebee on average stayed closer to the nest but showed a larger maximum distance from nest in comparison to the experienced bumblebees (Table S3), which practically did not venture further than the limits of the array (Fig. 2 in Lihoreau et al. (4)). This is consistent with the fact that during the first foraging bouts (most likely NNV bouts) a bumblebee exploits near-nest flowers but also perform flights much beyond the 50 m required to reach the next closest flower in the pentagonal array (Fig. S13B, Table S3). These structure of short and extra-long alternated flights is reminiscent of learning flights (20,21) and orientation flights (e.g. 22) that bees perform to acquire information about the visual environment surrounding important targets such as the nest and rewarding feeding locations.

**REFERENCES**

1. Riley JR, Smith AD, Reynolds DR, Edwards AS, Osborne JL, Williams IH, et al. Tracking bees with harmonic radar. Nature. 1996;379:29-30.

2. Burnham KP, Anderson DR. Model Selection and Multimodel Inference. A Practical Information-Theoretic Approach. Second edition ed. New York: Springer-Verlag; 2010.

3. Clauset A, Shalizi CR, Newman MEJ. Powerlaw distributions in empirical data. SIAM Rev. 2009;51:661–703.

4. Lihoreau M, Raine NE, Reynolds AM, Stelzer RJ, Lim KS, Smith AD, et al. Radar tracking and motion-sensitive cameras on flowers reveal the development of pollinator multi-destination routes over large spatial scales. PLoS Biol. 2012;10(9):e1001392.

5. Lihoreau M, Ings TC, Chittka L, Reynolds AM. Signatures of a globally optimal searching strategy in the three-dimensional foraging flights of bumblebees. Sci Rep. 2016;6:30401.

6. Szu H, Hartley R. Fast Simulated Annealing. Phys Rev Lett A. 1987;122:157-62.

7. Méndez V, Campos D, Bartumeus F. Stochastic foundations in movement ecology: Anomalous diffusion, front propagation, and random searches. Berlin Heidelberg: Springer-Verlag; 2014.

**Section S4. Perturbation analysis on transition probabilities**

Traplining bouts showed fairly constant values of H_trans._ around value 2.59 (less dispersion of the green points in Figures S14 and S15B). Importantly, an average value of 2.59 is obtained when pooling together Traplining synthetic bouts that minimize travel distances (N-1-2-3-4-5-N). As another reference, synthetic NNV bout minimizing travel distances (N-1-5-N) would have a much lower entropy H_trans_ = 1.585. In order to assess the impact of stochastic forces acting on these ideal Traplining bouts, we performed a perturbation analysis. We added a random, local perturbation (mean=0 and standard deviation range Stdv=[ 0.001-0.01]) to transition probabilities between flowers, using the ideal Traplining bout as a starting point (Fig. S16). The stochastic fluctuations applied to the ideal Traplining bout led to a significant decrease in the determinism and an increase in the mean turning angle, while little or no changes were observed in H_trans_ and bout size (Fig. S16). The values observed for a perturbation with a standard deviation of 0.01 were similar to those observed during the experiment for Route Development bouts (compare Figs 2A, 2B and 2D, and Table S1).


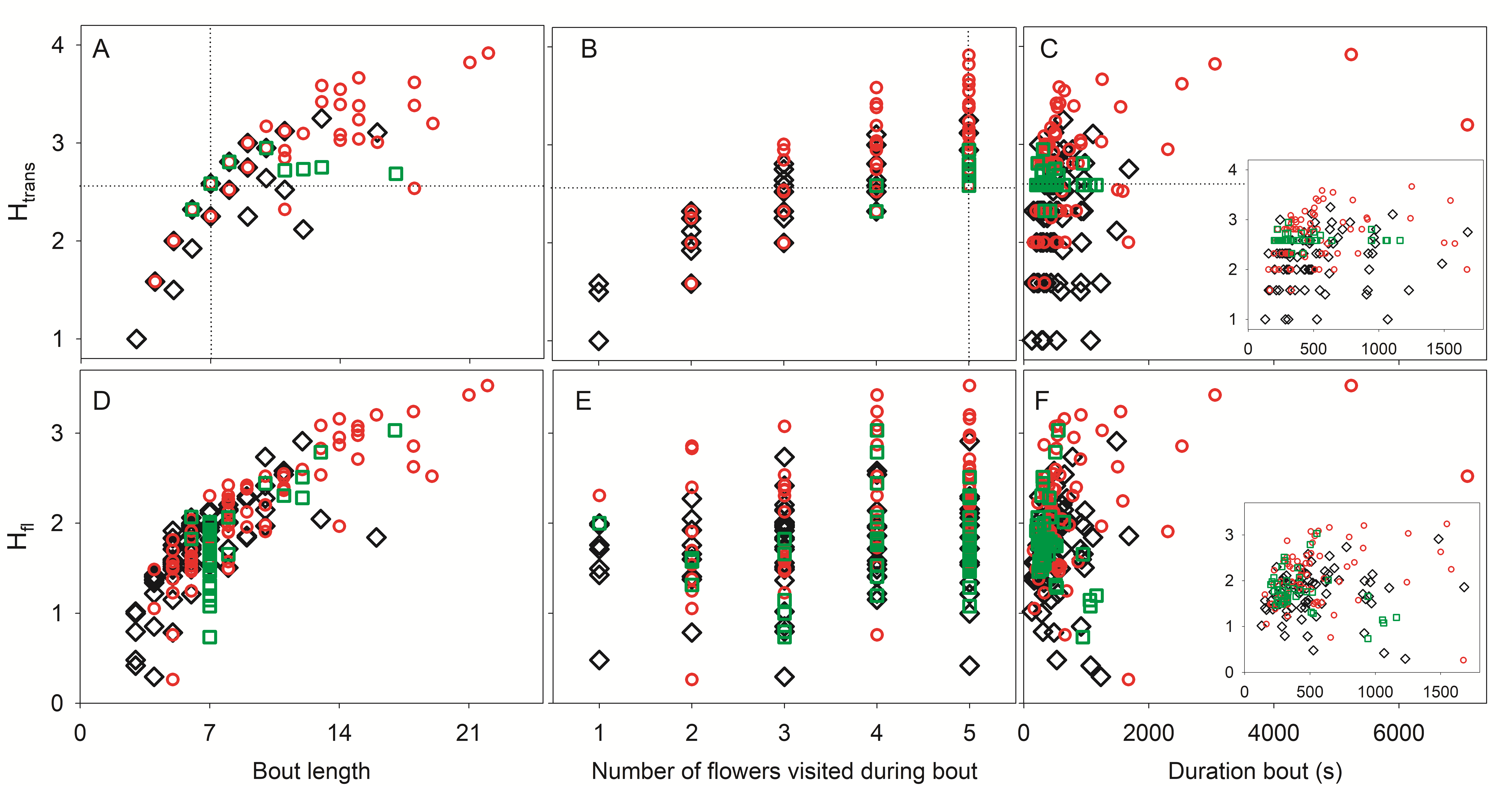


**Fig. S14. Entropy semantics.** Relationship between entropies A-C) H_trans_ or D-F) H_fl_ and the foraging bout variables: A,D) length of the flower visitation sequence, B,E) richness as number of different flowers visited during a bout (from 1 to 5), and C,F) duration of the foraging bout. Black diamonds, red circles and green squares represent NNV, Route Development and Traplining bouts, respectively. To improve visualization, insets on panels C and F show the subset of foraging bouts lasting from a few to 2000 s.

**Section 5. Additional Supplementary Figures.**





**Fig. S15. Temporal evolution of behavioural strategies and flight time entropy from naive to experienced bumblebees.** A) Time series of fluctuations between behavioural strategies Near-Nest Visits (NNV), Route Development (R) and Traplining (T) throughout testing of each of the 7 bumblebees evaluated. B) Return plots of entropy H_fl_ estimated in two successive bouts (“b”) of the same behavioural strategy: B) NNV, C) Route Development and D) Traplining. Note the oscillatory nature of H_fl_ during NNV and Route Development bouts where higher values are often followed by lower entropies. In NNV and Route Development bouts oscillations occur mainly between 0.3 and 2.8, and 1.1 and 3.7, respectively. In contrast with H_trans_ (Fig. 3 in main text, Traplining bouts show a larger variability with entropy values in the range 1-3.

D

C

A

B


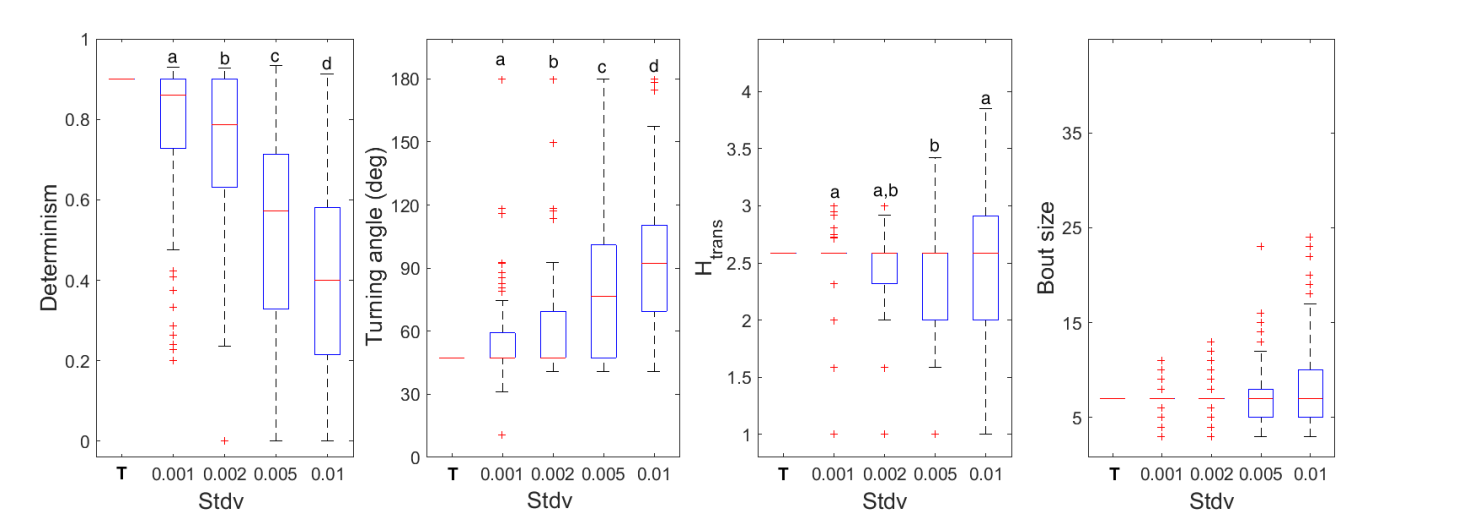


**Fig. S16. Perturbation analysis: effect on a selected set of variables of adding stochasticity on an ideal Traplining bout.** For perturbation analysis a random, local perturbation (mean=0 and standard deviation range Stdv=[0.001-0.01]) was added to the transition probabilities between flowers starting from the ideal traplining bout (T). Boxplots of A) Determinism, B) Mean turning angle, C) Entropy H_trans_, and D) length of flower visitation sequence of a foraging bout as a function of the standard deviation (Stdv) added. For each value of Stdv we did the estimates over 196 foraging bouts. An ideal traplining bout (a five flower visitation sequence minimising overall travel distances) was defined N-1-2-3-4-5-N. Strategies that do not share the same letter differ by p < 0.05 (Kruskal Wallis test).

**

**

**Fig. S17. Bar histograms of the length of the flower visitation sequence**. Bars represent the probability of performing a given number of flower visits during a foraging bout (bout length). The probability of immediate revisits (black) was estimated as the probability of performing an immediate revisit during a foraging bout, multiplied by the probability of performing a foraging bout of that length. Probability of non-immediate revisits (light grey) was estimated as the probability of returning to a flower that had been previously visited during a bout of a given length (but not consecutively after leaving that same flower), multiplied by the probability of performing a bout of that length. Probability of visiting a flower not previously visited during a foraging bout of a given length multiplied by the probability of performing a foraging bout of that length. **Inset:** Histogram of foraging bout durations. For visualization purposes, a maximum value of 3400 s was used on the x-axes.

**Fig. S18. Erratic temporal sequences by single bees on the t-Stochastic Neighbouring Embedding (t-SNE) space.** The main domains associated with the three behavioural strategies: Near-Nest Visits (NNV, black circles), Route Development (red circles), and Traplining (green circles) are represented. Blue lines indicate the temporal sequence through the t-SNE space as they gained experience, from naive at the beginning of testing to experienced towards the end of the observations.


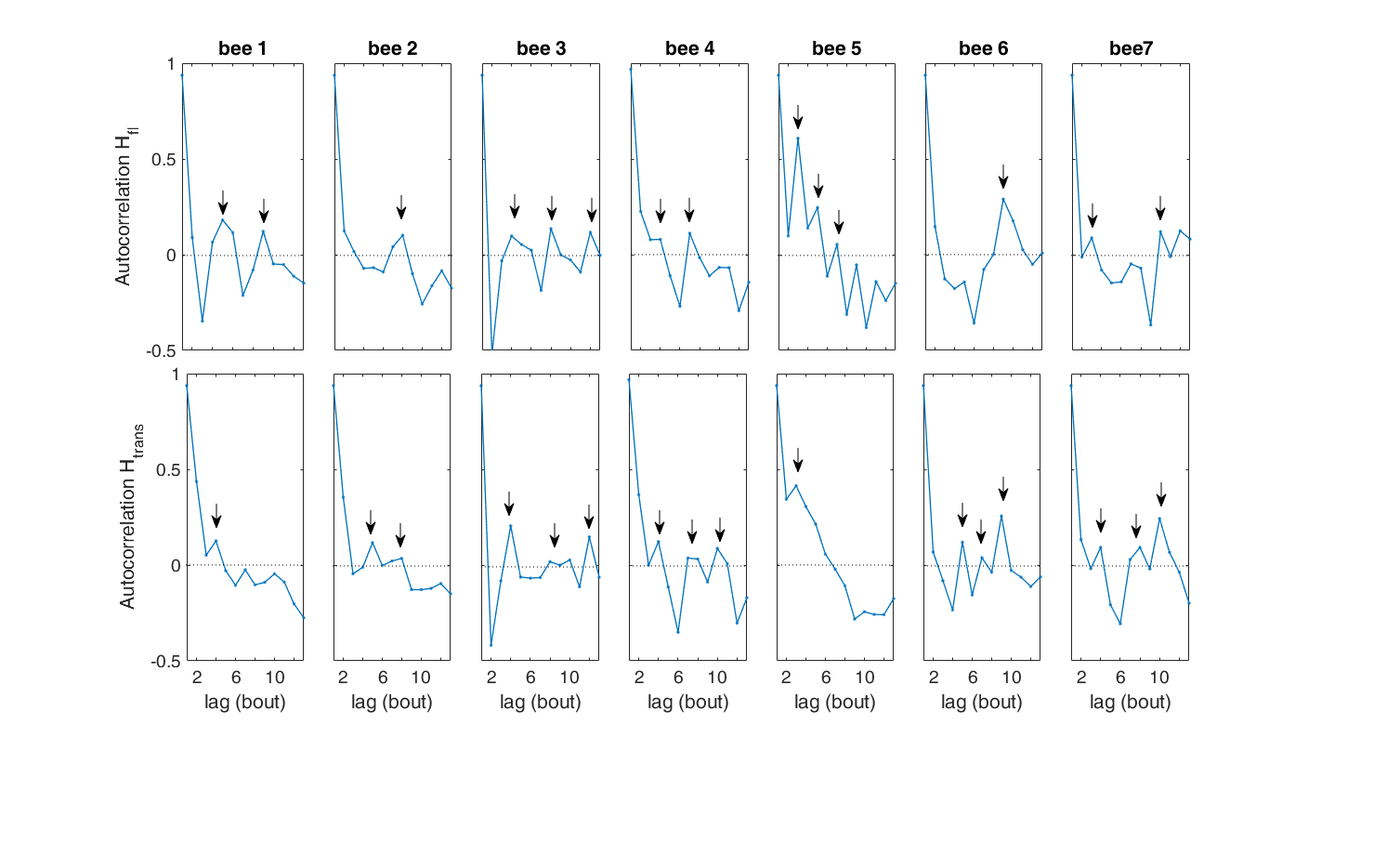


**Fig. S19. Autocorrelation plots of both H_ft_ and H_trans_ for each of the 7 bumblebees tested**. We indicate with arrows the fact that negative and positive correlation oscillations are of about lag 2. This means that every few foraging bouts the bees shift entropy values, from high to low and vice versa.


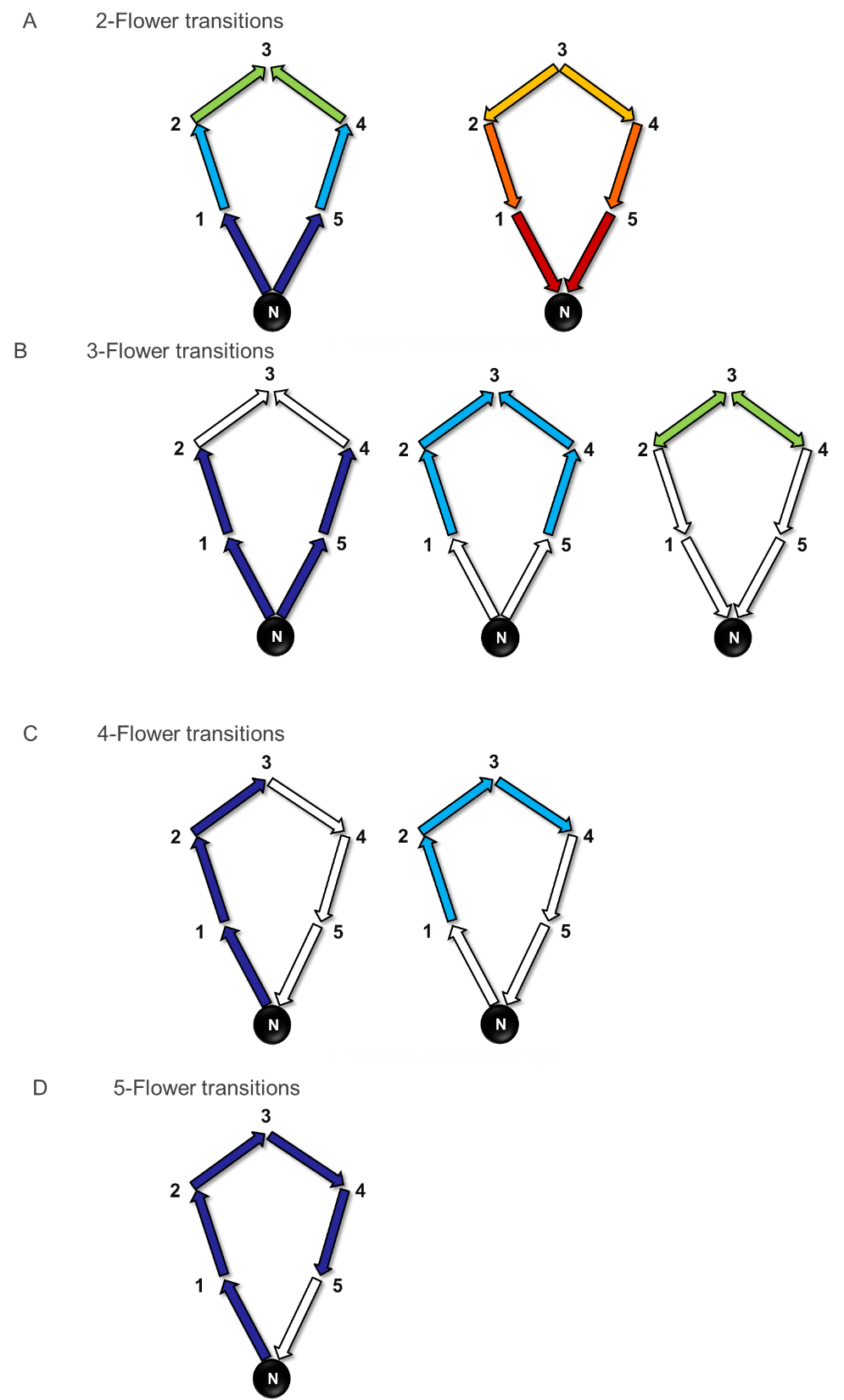


**Fig. S20.** **Classification of transitions according to symmetries**. We assumed symmetrical flower transitions to be equivalent in terms of path information. A) Symmetrical 2-Flower transition of types 1 (dark blue), 2 (light blue), 3 (green), 4 (yellow), 5 (orange) and 6 (red). B) Symmetrical 3-Flower transition of type 1 (dark blue), type 2 (light blue), and type 3 (green). N: nest, 1-5: flowers 1 through 5. C) Symmetrical 4-Flower transition of type 1 (dark blue) and type 2 (light blue), and D) Symmetrical 5-Flower transition. The arrows indicate the direction of the transition, while the double green arrows indicate the equivalence between the transition 2-3-4 and 4-3-2. In order to simplify the representation, in C and D we only show one direction.

**Table S4.**  Transition probabilities between flower estimated for Near-Nest Visits (NNV), Route Development and Traplining behavioural strategies as determined by t-Stochastic Neighbouring Embedding analysis (t-SNE).

|  | NNV |  | Route Development |  | Traplining |  | p-value |
| --- | --- | --- | --- | --- | --- | --- | --- |
| Symmetrical 2- Flower Trans. of Type 1 (%) | 20^a^ (13; 25) |  | 13^b^ (8; 17) |  | 17^b^ (14; 17) |  | <0.001 |
| Symmetrical 2- Flower Trans. of Type 2 (%) | 0^a^ (0; 20) |  | 14^a,b^ (0; 20) |  | 17^b^ (17; 17) |  | 0.01 |
| Symmetrical2- Flower Trans. of Type 3 (%) | 0^a^ (0; 0) |  | 14^b^ (8; 20) |  | 17^b^ (17; 17) |  | <0.0001 |
| Symmetrical 2- Flower Trans. of Type 4 (%) | 0^a^ (0; 0) |  | 11^b^ (0; 14) |  | 17^c^ (17; 17) |  | <0.0001 |
| Symmetrical 2- Flower Trans. of Type 5 (%) | 0^a^ (0; 17) |  | 0^b^ (0; 8) |  | 17^c^ (17; 17) |  | <0.0001 |
| Symmetrical 2- Flower Trans. of Type 6 (%) | 14^a^ (0; 25) |  | 0^b^ (0; 11) |  | 17^a^ (17; 17) |  | <0.0001 |
| 3-Flower Trans. Type 1 (%) | 0 (0; 0) |  | 1 (0; 17) |  | 20 (20; 20) |  | - |
| 3-Flower Trans. Type 2 (%) | 0 (0; 0) |  | 0 (0; 14) |  | 20 (20; 20) |  | - |
| 3-Flower Trans. Type 3 (%) | 0 (0; 0) |  | 0 (0; 0) |  | 20 (20; 20) |  | - |
| 4-Flower Trans. Type 1 (%) | 0 (0; 0) |  | 0 (0; 06) |  | 25 (22; 25) |  | - |
| 4-Flower Trans. Type 2 (%) | 0 (0; 0) |  | 0 (0; 0) |  | 25 (25; 25) |  | - |
| 5-Flower Trans. (%) | 0 (0; 0) |  | 0 (0; 0) |  | 25 (22, 25) |  | - |
| Near Trans. from Flower 1 (%) | 17 ^a^ (0; 25) |  | 7 ^b^ (0; 14) |  | 17 ^a^ (17; 17) |  | <0.0001 |
| Near Trans. from Flower 2 (%) | 0^a^ (0; 0) |  | 9^a^ (0; 14) |  | 17^b^ (17; 17) |  | <0.0001 |
| Near Trans. from Flower 3 (%) | 0^a^ (0; 0) |  | 11^b^ (0; 14) |  | 17^c^ (17; 17) |  | <0.0001 |
| Near Trans. from Flower 4 (%) | 0^a^ (0; 0) |  | 10^b^ (0; 14) |  | 17^c^ (17; 17) |  | <0.0001 |
| Near Trans. from Flower 5 (%) | 0^a^ (0; 20) |  | 8^a^ (0; 14) |  | 17 ^b^ (14; 17) |  | 0.005 |

Median (Q1; Q3) ^a-c^ Strategies that do not share the same letter differ by p < 0.05 (Kruskal Wallis). * Some of these variables are shown in Table 1, but are repeated here for completeness.

**Fig. S21. Routine movement behaviour analysis.** Time series of fluctuations in routine movement behavior (R) throughout route learning of each of the 7 bumblebees evaluated. Routine movement behavior is defined as R=1-H_p_, where H_p_ is the first order conditional entropy with p=1. H_p_ was estimated as proposed by Riotte-Lambert et al. (2017, Behavioral Ecology 28: 280-287) and using a moving window which included the sequence data of either 3 (left panel) or 5 consecutive bouts to smooth out the R curves. The routine movement behaviour is estimated as R=1- H_p_, where H_p_ is the pth order conditional entropy, H_p_, which quantifies the average uncertainty about the next symbol, knowing the string S_p_ = ...s_l_s_k_s_j_ of the p ≥ 1 previously occurring ones, corresponding to visited sites in the present context, ranked from the oldest to the current one: $H_{p}=-\sum_{.}^{*} P\left( \boldsymbol{S}_{\boldsymbol{P}} \right)\sum_{i=1}^{n} P\left( {s_{i}|\boldsymbol{S}}_{\boldsymbol{P}} \right) {log}_{n}\left[ P\left( {s_{i}|\boldsymbol{S}}_{\boldsymbol{P}} \right) \right]$ $=-\sum_{i=1}^{n} \sum_{.}^{*} P\left( \boldsymbol{S}_{\boldsymbol{P}}s_{i} \right){log}_{n}\left[ P\left( \boldsymbol{S}_{\boldsymbol{P}}s_{i} \right)/P\left( \boldsymbol{S}_{\boldsymbol{P}} \right) \right]$, where ∑* represents the summation for all possible strings of p sites successively visited. Note that the first-order conditional entropy values fluctuate over time, and in general decrease throughout route learning (frequency of traplining increases). It should be taken into consideration that the reliability of estimate could be low due to the short length of the sequences used in this study (a small number of flowers are visited per bout) as shown by (Riotte-Lambert et al. 2017).
